# Sexual ancestors generated an obligate asexual and globally dispersed clone within the model diatom species *Thalassiosira pseudonana*

**DOI:** 10.1038/s41598-018-28630-4

**Published:** 2018-07-12

**Authors:** Julie A. Koester, Chris T. Berthiaume, Naozumi Hiranuma, Micaela S. Parker, Vaughn Iverson, Rhonda Morales, Walter L. Ruzzo, E. Virginia Armbrust

**Affiliations:** 1 University of North Carolina Wilmington, Department of Biology and Marine Biology, Wilmington, NC 28403 USA; 2 University of Washington, School of Oceanography, Seattle, WA 98195 USA; 3 University of Washington, School of Computer Science and Engineering, Seattle, WA 98195 USA; 4 University of Washington, eScience Institute, Seattle, WA 98195 USA; 5 University of Washington, School of Medicine, Genome Sciences, Seattle, WA 98195 USA; 6 Fred Hutchinson Cancer Research Center, 1100 Fairview Avenue North, Seattle, WA 98102 USA

## Abstract

Sexual reproduction roots the eukaryotic tree of life, although its loss occurs across diverse taxa. Asexual reproduction and clonal lineages persist in these taxa despite theoretical arguments suggesting that individual clones should be evolutionarily short-lived due to limited phenotypic diversity. Here, we present quantitative evidence that an obligate asexual lineage emerged from a sexual population of the marine diatom *Thalassiosira pseudonana* and rapidly expanded throughout the world’s oceans. Whole genome comparisons identified two lineages with characteristics expected of sexually reproducing strains in Hardy-Weinberg equilibrium. A third lineage displays genomic signatures for the functional loss of sexual reproduction followed by a recent global colonization by a single ancestral genotype. Extant members of this lineage are genetically differentiated and phenotypically plastic, potentially allowing for rapid adaptation when they are challenged by natural selection. Such mechanisms may be expected to generate new clones within marginal populations of additional unicellular species, facilitating the exploration and colonization of novel environments, aided by exponential growth and ease of dispersal.

## Introduction

Sexual reproduction is presumed to have arisen in the last common ancestor of eukaryotes^1,2^ and subsequently was inherited throughout the eukaryotic domain. Recombination and segregation primarily benefit a population by increasing the fitness of individuals in the next generation or by providing phenotypic variance to drive adaption through selection over longer timescales^3^. A consequence of sex is a regression to a mean phenotype, through repeated reshuffling of allele combinations. Multiple taxa evade taxon- and life history-dependent costs of sexual reproduction^4^ through partial or complete loss of typical sexual pathways^5,6^. Although particularly fit asexual progeny may arise from sexual populations and thrive^7^, they are generally expected to have limited phenotypic versatility and thus to be evolutionarily short-lived^8^. Large gaps in our understanding of the life histories of the diverse unicellular organisms that dominate eukaryotic lineages^9^ complicate efforts to understand population-to-ecosystem-level dynamics.

Diatoms are a ubiquitous group of unicellular diploid algae whose marine populations link the biogeochemical cycles of carbon, silicon, and nitrogen through an ∼20% annual contribution to global primary production^10,11^. Despite the potential for panmictic gene flow facilitated by aquatic dispersal, diatoms can maintain geographically distinct populations^12^. Similar to other microbial eukaryotes, diatoms are facultatively sexual. They undergo extended periods of asexual (mitotic) reproduction, with a unique result that the average cell size of the diatom population decreases due to constraints of the silica cell wall. Diminished cell size and environmental cues^13–15^ trigger periodic sexual events, which restore cell size (*inter alia*). A few species, including *T. pseudonana*^16^, are able to escape the fate of mitotic size reduction. Importantly, facultative sexuals can capitalize on the fittest genotype-phenotype combinations during the exponential, asexual phase of population growth^17^ while retaining the ability to purge deleterious mutations and respond to environmental change through sexually derived diversification.

To explore the biogeographic diversity of *T. pseudonana*, we sequenced the whole genomes of isolates collected from the Adriatic Sea and the Atlantic, Indian, and Pacific Oceans, in addition to re-sequencing the original reference isolate CCMP 1335 isolated from the Western Atlantic (Supp. 1, Figs S1, S2). Aligned nucleotides from each isolate matched the 32 Mb reference genome^18^–a Sanger-derived, majority-rules, haploid sequence–at more than 99% of positions. The isolates have similar relative DNA content (ploidy) (Supp. 2.1, Fig. S3), while exhibiting ∼6-fold variation in estimated 18S rDNA copy numbers (Table 1, Supp. 2.2). Partial chromosomal deletions within each isolate leave them hemizygous at ∼1–6% of positions in the haploid genome; the extent of these deletions strongly correlates with time since isolation (*ρ* = −0.94, Supp. 2.3, Fig. S4), suggesting that such deletions accumulate and are better tolerated in culture than in the wild. These features allow the seven isolates to be readily distinguished from one another. Despite the high levels of sequence identity, the seven isolates group into high (H) or low (L) diversity subtypes relative to the reference sequence (Table 1). The two H-isolates, one from the Adriatic Sea (CCMP 3367) and one from the North Atlantic (CCMP 1013), displayed twice as many single nucleotide polymorphisms (SNPs) (Methods, Supp. 3) as the five L-isolates. The H-isolates also displayed ∼2 orders of magnitude more isolate-specific, private SNPs than the L-isolates (Table 1).

**Table 1:** General characteristics for the seven re-sequenced *T. pseudonana* isolates.

CCMP ID^a^ - Location	Cov^b^	18S CN^c^	SNPs^d^	Clade^e^	SNPs Shared with 1335^f^	Private SNPs^g^	Crossovers per Kb^h^
1335- New York, USA	108	8.2	180 K	L	100.0%	620	—
1007- Virginia, USA	37	26.7	183 K	L	92.8%	321	0.032
1012- Perth, Australia	71	21.4	186 K	L	94.3%	611	0.040
1015- Washington, USA	62	11.7	190 K	L	97.1%	2,070	0.040
1014- N. Pacific Gyre*	33	4.5	150 K	L	78.2%	559	0.086
1013- Wales, UK	70	9.7	304 K	H	61.7%	93,481	2.065
3367- Venice, Italy	64	9.0	291 K	H	59.6%	84,335	2.838

^a^National Center for Marine Algae and Microbiota (NCMA) identification number.

^b^Mean genome sequence coverage.

^c^Estimated 18S ribosomal DNA Copy Number (Supp. 2.2).

^d^Within-strain heterozygous positions (single nucleotide polymorphisms) based on refined SAMtools calls (Methods, Supp. 3); K = ×1000.

^e^Low (L) or High (H) diversity clade, based on total number of SNPs.

^f^Percent of refined SNPs in given isolate that are also present in CCMP 1335.

^g^Number of SNPs uniquely found in the given isolate.

^h^Estimated crossover density per kilobase relative to CCMP 1335 (Supp. 5.4).

*DNA sequence for 1014 was generated on a different sequencing platform.

**Figure 1:**
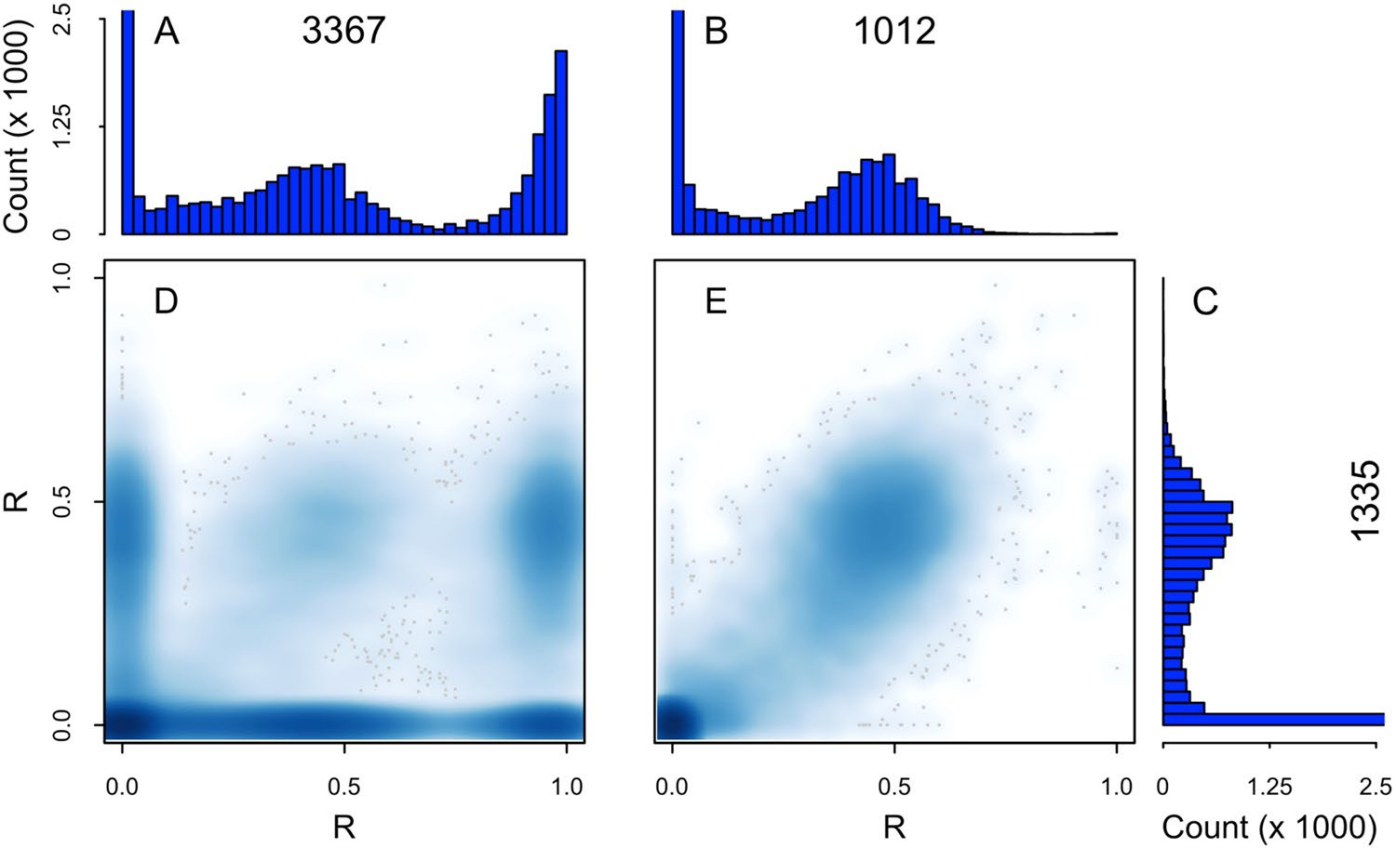
Distributions of the fraction of non-reference nucleotides per given genomic position (R) in L- and H-isolates. (**A**) H-isolate CCMP 3367, (**B**) L-isolate CCMP 1012, and (**C**) reference strain L-isolate CCMP 1335. Histograms are truncated at count 2,500 to highlight non-zero fractions within the distributions. (**D**,**E**) Pairwise comparisons of R-values from CCMP 1335 against corresponding positions in CCMP 3367 and CCMP 1012. For visual clarity, all plots were restricted to 35,291 positions on Chromosome 1 where none of the seven isolates had extreme read coverage and at least one had R ≥ 0.1 (Supp. 4). Darker shades of blue reflect a higher density of points; discrete gray points are outliers in regions of low density that surround higher density regions; e.g., the ≈20 discrete points near *x* = 1.0, *y* = 0.5 in panel E are the only positions (of 35,291) falling in this region.

The observed sequence variation between the L- and the H-isolates suggested a divergence in evolutionary trajectories potentially rooted in reproductive mode rather than reduced gene flow via isolation-by-distance. The diploid nature of diatoms allowed us to evaluate the extent of allelic variation within isolates by calculating a per-position statistic, *R*, that estimates the fraction of non-erroneous sequence reads bearing a nucleotide that is different from the reference at a given genome position (Supp. 4). We calculated the R statistic after taking into account mapping bias that occurred when sequence reads were aligned to the reference genome (Supp. 4.1, Fig. S5). In all isolates, nearly all aligned reads are identical to the reference sequence at the vast majority of positions (*R* ≈ 0), as expected at homozygous positions. All isolates also contain a subset of positions (>10^5^ in all cases) where *R* is near 0.5 (Fig. 1A–C, Supp. 4.2, Fig. S6), as is expected at heterozygous positions. A third subset of positions with *R* ≈ 1 is found almost exclusively in the H-isolates (>10^5^ in both, an order of magnitude more than in any L-clade member) as expected for positions that are homozygous for a non-reference nucleotide. Additionally, observed heterozygous positions in the reference isolate, CCMP 1335, often segregate into homozygous reference or homozygous non-reference sites in the H-isolates, for example in CCMP 3367 near coordinates (*R*_3367_, *R*_1335_) = (0.0, 0.5) and (1.0, 0.5) (Figs 1D; S6). Furthermore, the *R* distributions of both H-isolates fit the theoretical model predicated on Hardy-Weinberg equilibrium (HWE), specifically, exhibiting the expected 2:1 ratio between the numbers of heterozygous to homozygous non-reference positions (Supp. 5.2, Fig. S7). In contrast, the R-distribution of each L-isolate is essentially identical to that of CCMP 1335 (Figs 1B,C, S6), with nearly all SNPs shared among the L-isolates, and rare segregation of heterozygous positions (Figs 1E, S6). The frequencies of heterozygous positions also allowed us to confirm that each isolate comprises a single genotype (Supp. 5.3, Fig. S8). We predict that natural populations of H-isolates retain typical life histories in which sexual reproduction occurs periodically, whereas an alternative strategy, outlined below, appears to be employed by the L-isolates.

We performed 3 independent, quantitative analyses, each of which indicates that the L-isolates are not in HWE and collectively provide strong evidence that they represent a single mitotic lineage. First, we estimated the density of crossover sites between each pair of isolates (Supp. 5.4, Table S2). Our analyses estimate a low but non-zero density of crossover sites among L-isolates. In contrast, the density of crossover sites between L/H pairs and the H/H pair is an order of magnitude greater than between pairs of L-isolates (Supp. 5.4, Tables 1, S2). The number of false discoveries of crossover sites is difficult to estimate, but our analysis suggests that artifacts may inflate the L/L estimates, whereas the dichotomy of L/L pairs versus H-paired estimates remains robust. Second, in each L-isolate we find 1–2 orders of magnitude fewer (1,829–10,091) homozygous non-reference positions than predicted (∼90,000) based on the observed number of heterozygous positions. Third, assuming HWE, the chance that a SNP found in any L-isolate, e.g., the least heterozygous (CCMP 1014), is simultaneously found in the other four L-isolates is at most (1/2)^4^ = 1/16 (Supp. 5.5); however, more than 78% of its 89,184 SNPs (unrefined; Methods) have this property. This high level of agreement is essentially impossible under HWE; a set of only 24 randomly selected SNP positions (one per chromosome to avoid linkage) has less than a 6.94 × 10^−10^ chance of arising under the 1/16 HWE model (Supp. 5.5). Note that a *single* meiotic event along the ancestral lineage joining two isolates, even via selfing, would be expected to generate ∼90,000 homozygous non-reference positions and remove a large fraction of the shared SNPs. Collectively, the sharp reduction in recombination, scarcity of homozygous non-reference positions, and high SNP-identity among the L-isolates suggests that the globally distributed L-clade descended from a single ancestral genotype exclusively through mitotic reproduction.

Exclusively asexual reproduction is at odds with the general trend of cell size reduction noted earlier for diatoms. Cell diameter in *T. pseudonana* CCMP 1335 is remarkably stable in culture, possibly due to morphological characteristics (e.g., open girdle bands) that allow expansion in cell girth^16,19^. This phenomenon is unlikely to be restricted to *T. pseudonana* as other *Thalassiosira* species share this morphological trait^20^. Additionally, several large diatoms are known to undergo vegetative cell enlargement^21^, likely aided by girdle bands that are more aptly described as scales. Escape from mitotic cell size reduction can rescue cells that are too small and missed cues for sexual reproduction. Such escapes also allow for the transition to a purely asexual life history. *Thalassisoria pseudonana* has the morphological prerequisites for such a transition, but it is not unique among diatoms in exhibiting this potential.

**Figure 2:**
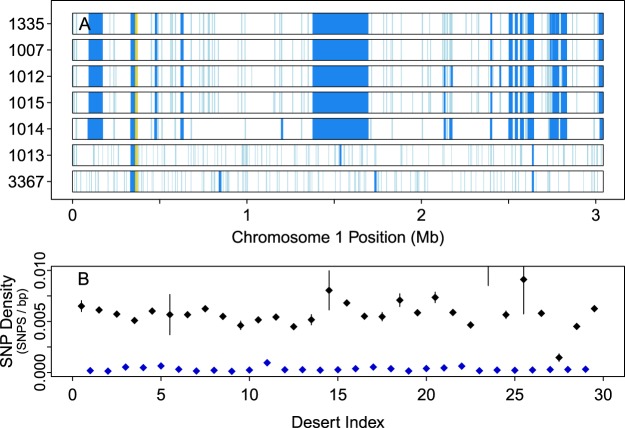
Loss of heterozygosity in *T. pseudonana* isolates. (**A**) Distributions of significantly low SNP regions (“deserts”; light blue: length <10 Kb; dark blue: >10 Kb) determined using a negative binomial model (Methods) across the 3 Mb of Chromosome 1 for the seven *T. pseudonana* isolates. The gold region near 0.3 Mb is a gap of known size in the reference sequence. The large region centered near 1.5 Mb is a 320 Kb desert present in all L-isolates but neither H-isolate. (**B**) SNP densities in the 29 deserts that span at least 50 Kb of the CCMP 1335 genome (blue) and the thirty regions surrounding these deserts (including deserts shorter than 50 Kb; black). “Density” (*p*) in a region of length *L* having *n* SNPs is calculated and plotted as *p* ± 2σ, where *p* = *n*/*L*, and ${\rm{\sigma }}=\sqrt{p(1-p)/{L}}$.

A substantial loss of heterozygosity marks a recent emergence of the L-clade and suggests a mechanism for a shift in life history strategy. Each isolate possesses hundreds of low-SNP-density regions (“SNP deserts”) across their chromosomes. Most span a few kilobases, comparable to the lengths of genes, suggesting purifying selection as a driver (Supp. 6, Fig. S9). In the H-isolates, the longest SNP desert is ≈45 Kb in length. In contrast, each L-isolate has at least 29 longer SNP deserts, ranging in size from ≈50 to 320 Kb that together span nearly 9% of each genome in noncontiguous blocks across multiple chromosomes. This pattern eliminates mitotic forms of recombination as potential mechanisms driving these large-scale loss of heterozygosity (LoH) events^22^. Most of these long deserts share essentially identical boundaries across the L-isolates (Fig. 2A, Supp. 6). For example, exact left and right boundaries of the 320 Kb desert on Chromosome 1 are each shared by 3, albeit different, L-isolates (Table S3). Furthermore, this large desert contains fewer than 75 SNPs per L-isolate, roughly 20-fold fewer than expected, and 50-fold fewer than observed in the same region in either H-isolate. We evaluated the relative ages of the large SNP deserts in the L-isolates. Assuming uniform mutation rates, neutral selection, and a SNP-free origin of each large desert, older deserts would have accumulated more SNPs per base pair than younger ones. We found that these large, low-heterozygosity regions have similar SNP densities that are ≈20-fold lower than intervening regions, implying an evolutionarily recent and nearly simultaneous LoH in the L-isolates (Fig. 2B). Gene conversion or selective sweeps would yield a spectrum of ages. In contrast, inbreeding, during a population bottleneck for example^23^, is consistent with the observed simultaneous LoH and seems the most likely mechanism for it. Observed heterozygote deficiency during diatoms blooms^24^ supports potential inbreeding within natural communities despite cell densities reaching thousands per liter of seawater.

Asexuality may arise if fertilization results in the homozygous pairing of preexisting deleterious alleles for a key gene or genes required for sexual reproduction. For example, obligate parthenogenesis in the rotifer *Brachionus calyciflorus* is associated with a recessive allele that in homozygotes disrupts the response to sexual chemical cues without affecting the viability of the resulting asexual offspring^25^. In this light, we note that three of 33 annotated genes associated with sexual reproduction lie within SNP deserts in the L-isolates (two are located within the 320 Kb desert on Chromosome 1 and the third lies in a shorter desert on Chromosome 7). Two genes are homologs of the sexually induced genes (*SIG* *1* and *2*) that are putatively necessary for gamete recognition^26^ and are SNP-free in the L-isolates, as is a third gene encoding a homolog of the flagellar ribbon protein RIB43a in *Chlamydomonas reinhardtii*^27^ that, in diatoms, is likely required for sperm motility. In contrast, all three genes contain both synonymous and non-synonymous SNPs in the H-isolates relative to the reference isolate (Supp. 7, Table S4) that presumably reflect standing variation in the wild type sexual population. The majority of the genes required to induce and complete sexual reproduction remain unknown in diatoms; we speculate that homozygosity for one or more alleles such as these prevented the ancestors of the L-isolates from sexually reproducing, thus creating an asexual lineage.

We performed a suite of numerical simulations (Supp. 8), based on simple genetic models and realistic parameter choices, to explore whether the L-genotype is an *obligate* asexual. Given that the progression through meiosis and fertilization is a slower process than mitosis, an obligate asexual will outgrow its sexual counterparts during their infrequent meiotic interludes, resulting in sustained dominance of the asexual clone within a few centuries, even if it has no selective advantage other than the loss of sex (Fig. S10A). The simulations also support mitotic expansion of a *facultative* sexual genotype on a similar timescale when that genotype hosts an advantageous allele or combination of alleles that would be nearly neutral if disrupted by meiosis. The simplest example is an advantageous Mendelian recessive, i.e., an allele that is advantageous only when homozygous, which we modeled as an increase in time between meiotic events. These latter simulations, however, predict results *not* seen in our data. Specifically, as the advantageous genotype rises in frequency during the mitotic phase of growth, its occasional sexual offspring add its (post-recombination) haplotypes to the remainder of the population. The simulations indicate that sexual reproduction among these other lineages result in the reassembly of the advantageous allele combination (e.g., homozygous recessives), which leads to fixation of this/these allele(s) on new genetic backgrounds and extinction of the original L-genotype (Fig. S10B). In summary, the success of the clonal L-genotype is most readily explained by obligate asexuality. Existence of the H-isolates, and other sexual lineages that we assume occur in natural populations, depends on factors, such as potential local adaptions or geographic isolation^28^, that lie outside the scope of the models considered here.

**Figure 3:**
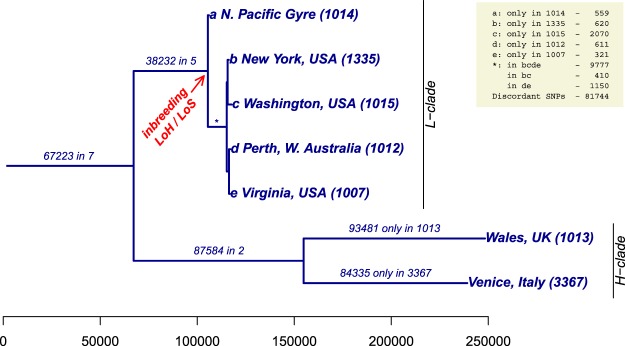
SNP sharing among the seven strains of *T. pseudonana*. Cladogram based on a total of 468,117 refined SNPs, accounting for 82% of SNP positions, identified across all 7 strains (Supp. 9). The tree is rooted at the common ancestor of all seven isolates, with each split selected to maximize the number of SNPs shared by isolates within each subtree and minimize sharing across subtrees. Internal branch lengths are the numbers of SNPs shared by all isolates in the subtree below that branch. Terminal branch lengths are the numbers of private SNPs found in each isolate. The red arrow reflects an inferred evolutionary event in which inbreeding resulted in a significant loss of heterozygosity (LoH) and concomitant loss of sexual reproduction (LoS) that founded the L-clade. Scale: number of SNPs.

Asexual lineages are expected to possess limited phenotypic versatility and thus to be evolutionarily short-lived^8^. However, given sufficient phenotypic plasticity, and especially given their exponential growth advantage, asexuals can found subpopulations in novel and marginal environments, potentially expanding the ecological niche, as predicted by the general-purpose genotype model, originally proposed in 1955 for plants^29^. The descendants of the inbred, asexual ancestor of the L-isolates successfully dispersed across the world’s oceans and are now genetically differentiated (Fig. 3, Supp. 9). We estimated the timing of the emergence of the L-clade by considering the time it would take an L-isolate to accrue its private SNPs. We assumed that *T. pseudonana* divides mitotically about once every two days and has a mutation rate of ≈10^−9^ mutations per base pair per division^30^. Given a 32 Mb haploid genome size, and assuming mutations are selectively neutral, the ≈2,000 private SNPs observed in CCMP 1015 (Table 1) could accumulate within 200 years of its separation from the other known L-clade members. The actual time since the emergence of the L-clade from its most recent common ancestor is longer, presumably a few hundred years, because the L-clade (like the H-clade) is likely underrepresented in our tree, and 200 years only estimates the length of one branch thereof. Circumnavigation of major ocean gyres via surface currents takes less than a decade^31^ and the exponential advantage of asexual growth suggests a natural route to the present-day distribution of the L-isolates; the potential for additional dispersal through human activity (e.g., ballast water) could have hastened their spread. With such a rapid dispersal potential, we also would expect the H-clade to be distributed globally. The prevalence of L- over H-isolates in publicly available culture collections may reflect an enhanced ability to acclimate to culture conditions rather than geographic exclusion of the H-clade. In short, global spread of the L-clade in a few hundred years is not implausible.

*Thalassiosira pseudonana* has a rich, decades-long history of research enabled by the ease with which it is cultured. We further evaluated the impact of culturing artifacts on our genomic data, especially given that the isolates have sustained genomic losses of hundreds of kilobases per decade in culture (Fig. S4). This particular artifact has the important benefit of alleviating concerns that cross-contamination explains the extensive shared genomic patterns among the isolates, especially the L-clade. Large-scale duplications have also occurred and also correlate with time in culture (data not shown). However, the bulk (>80%) of their diploid genomes are unaffected by such events, and the relevant patterns of sharing (within the L-clade) and differences (to/within the H-clade) are driven by this majority. Point mutations are also assumed to occur in culture, but would predominantly remain private to each cultured isolate, and we see at most a few thousand such events in any L-isolate. Hence, in-culture SNPs could neither dominate the ∼40,000 heterozygous (SNP) positions shared by the L-isolates, nor explain the systematic destruction of the ∼90 thousand homozygous nonreference positions expected if the L-isolates were sexually reproducing, nor the ∼3 million positions in shared SNP deserts. We note that neither overall SNP counts nor private SNP counts are correlated to the length of time each isolate was in culture. (In contrast, kilobase-scale duplications and hemizygous deletions, which *are* correlated to time in culture, are more likely to impact cell viability in the wild and are thus more likely to have been purged from the wild type population, and conversely more likely to have been tolerated in the less stressful culture environment.) In short, the results indicate that the genomically distinct L- vs H-patterns did not independently emerge in the seven separate cultures, but were shared by cells within natural populations that were isolated decades and thousands of miles apart.

The evolution of phenotypically plastic individuals depends upon the degree of plasticity in their ancestors. Within the unicellular eukaryote *Ostreococcus*, descendants with the greatest magnitude of adaptive response and range of phenotypic plasticity evolved from the most plastic lineages^32^. Six of the seven *T. pseudonana* isolates were collected from estuaries. Freshwater conspecifics of *T. pseudonana* have little to no variation from marine isolates at major bar-coding genes and the species is phylogenetically placed within the freshwater genus *Cyclotella*^33^ with *Thalassiosira pseudonana* originally described as *Cyclotella nana*^34^. Remarkably, CCMP 1014 was isolated from the open ocean. Although the sequence data derived from CCMP 1014 is technically different, we can still infer its membership in the L-clade. Similar to other L-isolates, it contains almost no homozygous non-reference sites, displays high concordance with L-isolate SNPs and SNP deserts (Figs 2A, S6), and has a low crossover rate (Table 1). Thus, it appears that CCMP 1014 breached the presumed ecological niche of *T. pseudonana* and adapted to the local environment as suggested by its physiological differences from CCMP 1335^35^. The exceptional salinity tolerance of the species *T. pseudonana* was reflected in the reference isolate CCMP 1335 (identified as 3 H) relative to ≈40 taxa^36^, suggesting that the L-isolates have the requisite phenotypic plasticity to predict further adaptation to novel environments. The colonization of an ecologically marginal habitat by CCMP 1014 is unlikely to be an isolated anomaly. Life histories of unicellular eukaryotes are dominated by an asexual phase that produces clonal free-living organisms. Our observation of the global distribution of a single unicellular and obligate asexual clone is unprecedented. Whether derivatives of this particular rapidly emergent clone will equally rapidly succumb to opportunistic pathogens, competition, or environmental change, or, on the other hand, firmly establish themselves as very long-term constituents of the marine ecosystem remains to be seen. In either case, our example of *T. pseudonana* may be generally applicable to other unicellular eukaryotes with rapid rates of asexual reproduction and potential for long-distance dispersal.

## Materials and Methods

### Data and code availability

Sequences are available through the NCBI SRA BioProject PRJNA376612. All custom scripts and associated data described here and in the supplemental information are available at https://github.com/armbrustlab/global_thaps_clones.git.

### Isolate selection and culturing

The seven globally distributed isolates of *T. pseudonana* (Fig. S1) are available from the National Center of Marine Algae and Microbiota (NCMA) as CCMP 1007, 1012, 1013, 1014, 1015, 1335, and 3367. We purchased the first six isolates from NCMA while the seventh isolate was provided by Raffaella Casotti (Stazione Zoologica Anton Dohrn) and subsequently deposited by us to the NCMA collection as CCMP 3367. All seven isolates were grown independently in f/2 medium with seawater from Puget Sound, WA, USA. Each isolate was made axenic by sorting (BD Influx) 5–10 cells per well into 96-well plates containing four-fold dilution series with starting concentrations of 4.48 × 10^−3^ M Penicillin and 1.11 × 10^−3^ M Streptomycin. Isolates were grown to 75 mL and tested for bacterial growth using bacto-peptone broth assays (50 µl culture:1 mL broth; two-day incubation). Isolates with negative assays were further tested by staining them with SYBR Green (1 × SYBR; 20 min incubation) and observing them with epifluorescence microscopy (Nikon Eclipse80i) using a 520 nm long-pass emission filter (Chroma 11000 series) at 100x magnification. Axenic cultures (15 L) of each isolate were grown to late exponential–early stationary phase in 20 L acid-cleaned Lightnin Biotech Mixers (Nalgene) under continuous light at 20 °C, except for CCMP 1335, which was grown on a 16:8 light/dark cycle at 13 °C. Isolates were filtered in 500 mL batches onto 47 mm, 0.4 μm Isopore HTTP filters, flash frozen in liquid N, and stored at −80 °C.

### DNA sequencing

We extracted DNA from each isolate using the DNeasy Plant Mini Kit (Qiagen). SOLiD 2 (Applied Biosystems) libraries of 35 bp fragments were constructed for CCMP 1335 (3 libraries) and CCMP 3367 (2 libraries) using 10 μg of DNA each. Mate pair libraries (SOLiD 2, 25 × 25 bp) were constructed for all seven isolates, each using 20 μg of DNA. Libraries were amplified on sequencing beads using ePCR. All libraries, except for CCMP 1014, were sequenced from 200–300 × 10^6^ beads on the SOLiD 2 platform, while the CCMP 1014 libraries were sequenced using SOLiD 3. Applied Biosystems protocols were followed for each phase of library preparation and sequencing. Sequencing was performed in 2008–2009.

### Initial Quality Control and Alignment

Native SOLiD 2 csfasta and qual read files were converted to FASTQ format using solid2fastq in the SEAStAR software package^37^. Low complexity reads were discarded using trimfastq in SEAStAR with the command:

$$\mathrm{trimfastq}\,-\mathrm{e3}\mathrm{.0}\,-124-\mathrm{p0}.0--\mathrm{no}-\mathrm{prefix}$$

where “-e 3.0” sets the entropy parameter to filter low complexity reads; “-l N” sets the minimum acceptable length of reads (N was set to 24 for 25 bp mate-paired reads and 34 for 35 bp single-ended reads); “-p 0.0” disables trimming by quality; and “--no-prefix” leaves read IDs unchanged.

Reads from each isolate were aligned to the nuclear reference genome of *T. pseudonana*, CCMP 1335, version 3.0 (Joint Genome Institute) using the BWA^38^ command:

$$\mathrm{bwa}\,\mathrm{aln}\,-\mathrm{k\; 2}\,-\mathrm{n\; .0}\mathrm{01}\,-\mathrm{c\; -}\mathrm{l\; 18}\,-\mathrm{t\; 8}$$

Resulting binary SAI files were converted to SAM format using the sampe and sampe subcommands of BWA for fragment and mate-paired runs respectively. Exact duplicate mate-paired alignments (but not unpaired fragments) were removed using the MarkDuplicates command of the Picard software package (http://broadinstitute.github.io/picard/).

### SNP Identification

SNP positions in each strain relative to the reference genome were identified using SAMtools^39^ and the following command pipeline:

$$\begin{array}{c}\mathrm{samtools\; mpileup}\,-\mathrm{C50}\,-\mathrm{A\; -B}\mbox{--}\mathrm{u\; -g}\,-\mathrm{q1}\,-\mathrm{f\; ref}\mathrm{.fasta}\mathrm{aln}\mathrm{.bam}|\backslash \\ \mathrm{bcftools\; view}\,-\mathrm{b\; -g}\,-\mathrm{t\; 0}\mathrm{.007}-|\mathrm{bcftools}\mathrm{view}\,-\,|\backslash \\ \mathrm{vcfutils}\mathrm{.plvarFilter}\,-\,\mathrm{D\; 10000} > \mathrm{variants}\mathrm{.vcf}\end{array}$$

where ref.fasta contains the nuclear genome of CCMP 1335, aln.bam is a file of aligned reads for an individual isolate, and variants.vcf is the output (SNP list) in variant call format. For bcftools view, the scaled substitution mutation rate (−t .007) was based on the estimated SNP rate in CCMP 1335^18^.

### Refining SNP Calls

SNP identification was refined to include positions where SAMtools called a SNP in one isolate but not in another, even though a significant number of reads for the same non-reference nucleotide were present. To be counted as a SNP in one isolate, the position was required to meet a set of criteria including minimum coverage and presence in another isolate (details and examples in Supp. 3).

### Quality Filtering

The varFilter variant calling algorithm (above) accounts for quality, coverage, and base calls, and so is somewhat protected from BWA’s bias against non-reference reads (Supp. 4.1, Fig. S5). However, this bias did affect the statistic R. To ameliorate this bias when calculating R, we used a subset of read data based on more stringent ABI quality metrics and custom scripts. Specifically, we translated the ABI SOLiD color space into nucleotide space and generated per position coverage tables for each isolate based on ABI quality scores. The command pipeline used mpileup from SAMtools plus custom scripts:

$$\begin{array}{c}\mathrm{samtools\; mpileup}\,-\mathrm{C50}\,-\mathrm{A\; -B\; -u\; -g}\,-\mathrm{q40}\mbox{--}\mathrm{fref}\mathrm{.fastaaln}\mathrm{.bam}|\backslash \\ \mathrm{python2}\mathrm{.7readCov2}\mathrm{.py}\\ \mathrm{R\; CMD\; BATCH}\,--\mathrm{no}-\mathrm{save\; buildTabs}\mathrm{.R}\end{array}$$

where ref.fasta and aln.bam are described above. The -q parameter sets a minimum Phred quality score of 40 for each position. readCov2.py is a custom Python script used to create quality filtered coverage tables for each strain (Table S1). buildTabs.R converted these outputs to a convenient format for downstream analysis by various custom R scripts.

### Calculating R

Reads that map to, but do not perfectly match, the reference sequence reflect a key signal of genetic diversity, mixed with technical sequencing errors. To summarize per-position nucleotide diversity, we extract two numbers: *rc* counts reads containing the reference nucleotide at that position and *nc* counts reads containing the most common non-reference nucleotide at that position. The ratio is calculated as:

1 $$R=\frac{nc}{nc+rc}$$

Positions with nonzero counts for two or more non-reference nucleotides are rare and most likely reflect technical errors instead of biological variation. The R statistic provides a simple way to remove some of the technical error. See the Supplemental Information (Section 4) for further discussion and additional pairwise plots of the R statistic.

### Identifying SNP “Deserts”

We quantified regions with significant loss of heterozygosity by employing a negative binomial model. Let 1/*p* be the strain-specific average distance in bp between heterozygous positions (SNPs). With the assumption that SNPs randomly and independently occur in the genome with per-base probability *p*, the distance from a given position to the *k* = 5th SNP in the 3′ direction (relative to the reference strand) follows a negative binomial distribution with parameters *p* and *k*. Letting *L* be the minimum distance between SNPs such that this probability is ≤10^−4^, all intervals of length >*L* in the genome containing fewer than *k* SNPs were identified and merged if they overlapped or were separated by ≤100 bp. Near the 3′ end of each chromosome, where there are j < 5 SNPs remaining, this procedure is modified to use the negative binomial with parameters p and j. The resulting regions were defined as SNP deserts.

## Electronic supplementary material

Supporting Information
