# Supplementary material for: Sexual ancestors generated an obligate asexual and globally dispersed clone within the model diatom species Thalassiosira pseudonana
Source: Sci Rep. 2018 Jul 12;8:10492. doi: 10.1038/s41598-018-28630-4 (PMC6043606; doi:10.1038/s41598-018-28630-4)
Supplement: Supplementary file 1 — Supporting Information [file 41598_2018_28630_MOESM1_ESM.pdf]

Supporting Information: Sexual ancestors generated an obligate asexual and globally dispersed clone within the model diatom species *Thalassiosira pseudonana*

Julie A. Koester[a,1], Chris T. Berthiaume[b], Naozumi Hiranuma[c], Micaela S. Parker[d], Vaughn Iverson[b,d], Rhonda Morales[b], Walter L. Ruzzo[c,e,f,1], and E. Virginia Armbrust[b]

[a]University of North Carolina Wilmington, Department of Biology and Marine Biology, Wilmington, NC, 28403, USA; [b]University of Washington, School of Oceanography, Seattle, WA, 98195, USA; [c]University of Washington, School of Computer Science and Engineering, Seattle, WA, 98195, USA; [d]University of Washington, eScience Institute, Seattle, WA, 98195, USA; [e]University of Washington, School of Medicine, Genome Sciences, Seattle, WA, 98195, USA; [f]Fred Hutchinson Cancer Research Center, 1100 Fairview Avenue North, Seattle, WA, 98102, USA

Abstract

This document presents the supporting information accompanying the manuscript **Sexual ancestors generated an asexual and globally dispersed clone within the model diatom species *Thalassiosira pseudonana.*** Supporting figures and cited methods are provided here; associated data and software are publicly available on Github[a].

[1]Questions concerning the methods and software can be addressed to: ruzzo@uw.edu
[a]`https://github.com/armbrustlab/global_thaps_clones`

1 Overview of the Experimental & Computational Methodologies — Analysis Workflow

Six isolates of *Thalassiosira pseudonana* originating from geographically distinct locations (Fig. S1) were obtained from the National Center of Marine Algae and Microbiota (NCMA); we received a seventh from Dr. Raffaella Casotti and provided it to NCMA as CCMP3367. Following in-house culturing, DNA obtained from 3 isolates was checked for genomic duplication, each isolate was sequenced with ABI's SOLiD sequencing technology, and independent analyses of the sequence data were conducted (Fig S2). Custom scripts and data analyses can be found where indicated in: `https://github.com/armbrustlab/global_thaps_clones`. Raw sequence data are available in the NCBI SRA BioProject PRJNA376612 (`https://www.ncbi.nlm.nih.gov/bioproject/PRJNA376612/`)

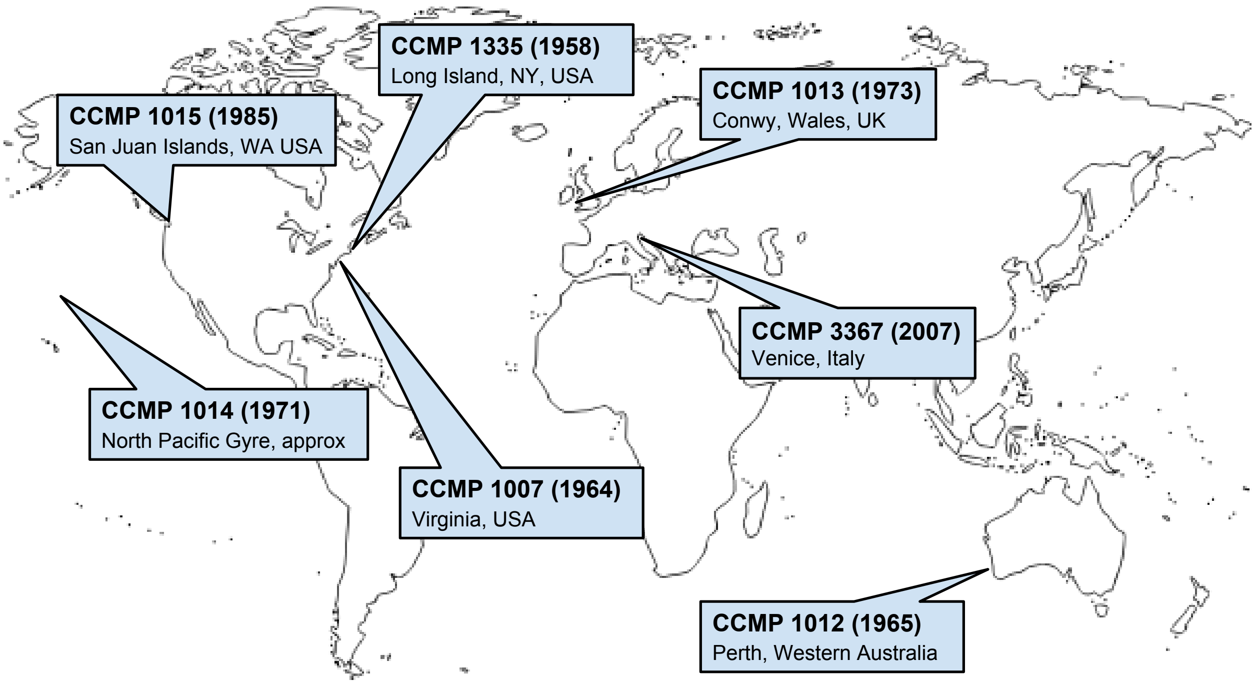

Figure S1. Geographic origin, CCMP identification number, and year of isolation for the 7 isolates of *T. pseudonana* used in this study.

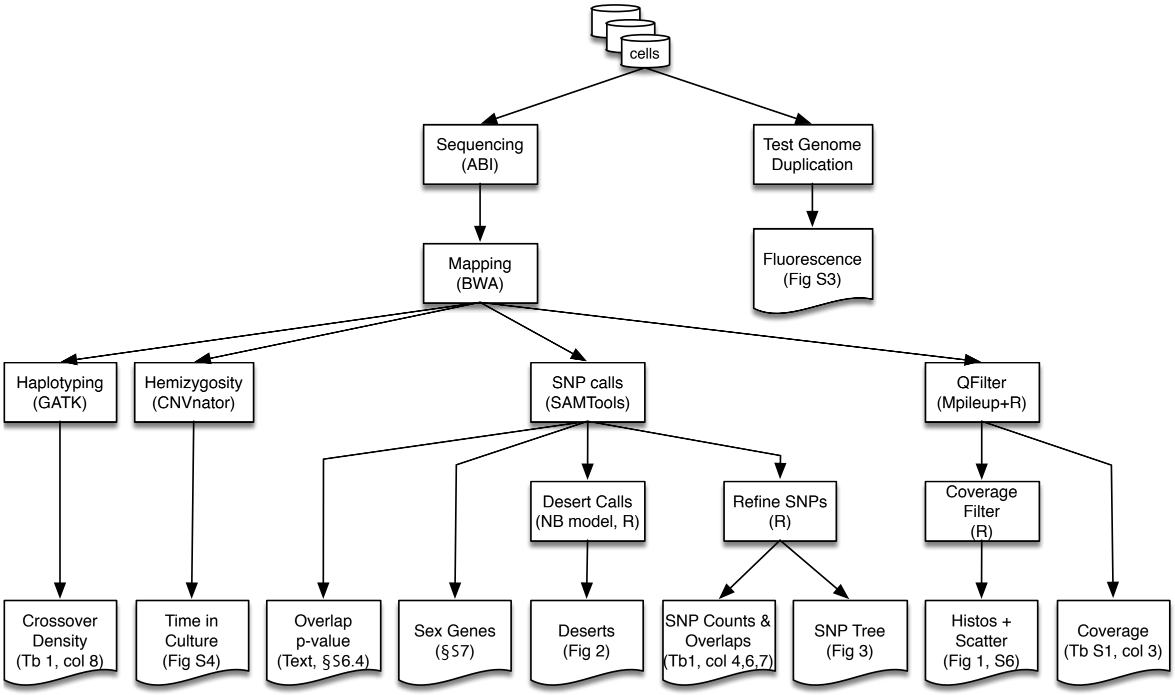

Figure S2. Workflow for experiments, computation, and comparative data analysis of 7 genomes of *Thalassiosira pseudonana*.

2 Confirmation that 7 isolates of *T. pseudonana* are genetically distinct

We determined that the 7 isolates of *T. pseudonana* were genetically distinct by quantifying copy number variation at the 18S locus and the extent of hemizygous deletions relative to the time each isolate has been in culture.

2.1 Test for Genome Duplication

Relative genome sizes of three *T. pseudonana* strains (CCMP 3367, CCMP 1013, and CCMP 1335) were determined by quantifying the fluorescence of SYBR-green stained nuclei with flow cytometry. Strains were grown at 13°C in a 16:8 hr light/dark cycle and sampled at the same time during the light period. Samples were fixed with 1% paraformaldehyde and 0.01% glutaraldehyde and incubated on ice for 20 minutes. Samples were diluted 3-fold with f/2 medium to maintain the analytical flow rate below 500 cell s^{-1}. Diluted samples were stained with 0.01% SYBR Green I (diluted with milliQ water) for 15 minutes at room temperature in the dark. Following the addition of fluorescent microspheres (1 µm, Invitrogen FluoSpheres) as an internal standard, stained samples were analyzed with a BD Influx flow cytometer (Senserion SLG-1430-480). Green fluorescence was measured in linear scale and at least 10,000 cells were collected per sample.

Data were obtained using the Spigot Operating Software version 5.0 (BD Biosciences) and analyzed using the `R` package `flowCore` version 1.30.7. Bimodal distributions of DNA were detected in each isolate (Fig. S3). Sampled cells were predominantly in the G_1 phase of the cell cycle represented by the large peak at $x \approx 15$. The peak near $x = 30$ is consistent with fewer cells sampled during $G_2 + M$. No difference in relative G_1 DNA content is observed among the different isolates. (Fig. S3).

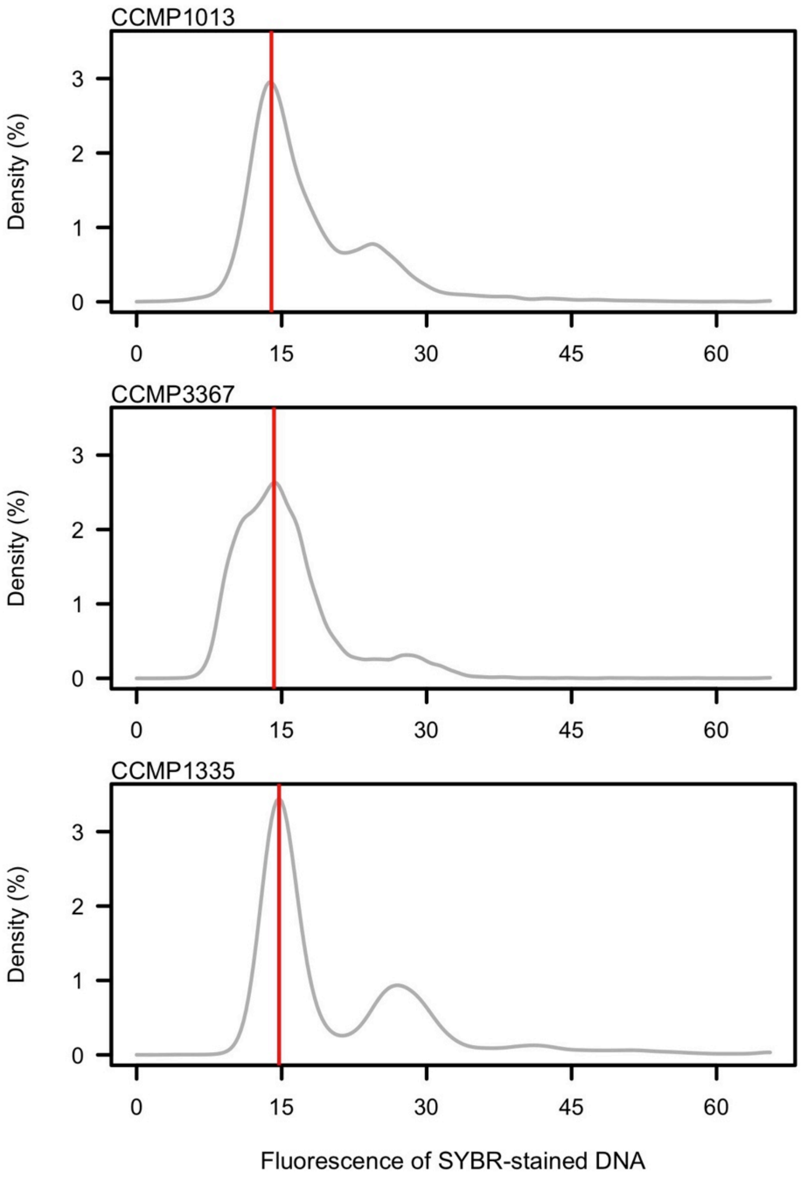

Figure S3. Relative DNA content of three *T. pseudonana* isolates: CCMP 1013, CCMP 3367, and CCMP 1335. The x-axes represent the relative fluorescence of SYBR-stained cells and the y-axes represent the proportion (density) of cells with a given fluorescence value, based on 250 bins each containing a small percentage of the approximately 10^4 cells sampled per isolate. The data were smoothed and plotted using the R function `density()` with default parameters and a Gaussian kernel.

2.2 Estimated Copy Number Variation of 18S Ribosomal rDNA

The 18S locus is represented as a single-copy gene in the CCMP 1335 reference genome sequence, although several tandem copies are present[1]. We used the ratio of average 18S read coverage to the genome-wide average read coverage as an estimate for the strain-specific copy number at the 18S locus. Estimated copy number varied from 5 to 27 across isolates, approximately (Table 1 main text). Although non-reference reads were observed at the 18S locus, no single nucleotide variants were called there by SAMtools, presumably because no single variant was repeated in sufficiently many copies.

2.3 Estimated Hemizygous Chromosomal Deletion

Read coverage also was used for *de novo* detection of other duplicated or deleted genomic regions within each isolate. Specifically, we used the software package `CNVnator`[2] to identify regions where coverage deviated significantly from the average across each isolate's genome. Commands can be found in the shell script in `global_thaps_clones/scripts/CNV`. The output of this shell script is a text file, `global_thaps_clones/data/cnv.txt`, giving the analysis results from `CNVnator`. (This procedure was not needed for the 18S locus, since its location was known *a priori*.)

Hemizygous regions (i.e., regions lacking one of the two copies expected in a diploid organism) were of particular interest. These were identified, based on the results from the `CNVnator` analysis, as those regions with approximately half of the isolate's genomic average read coverage. We processed the `CNVnator` output with the following gawk shell command:

```
gawk 'NR>1 && $7=="CNVnator" && $6=="False" && $8 >.3 && $8 < .7 \
&& substr($2,1,3)=="Chr" {x[$1]+=$5} END {for \
(strain in x) {print strain " " x[strain]}}' cnv.txt

   NR > 1                    # skips the header line
   $7 == "CNVnator"          # only consider CNVnator features
   $6 == "FALSE"             # feature isn't flagged as suspect
   $8 >= .3 && $8 <= .7      # feature has near 0.5 coverage
   substr($2,1,3) == "Chr"   # ignore non-chromosomal contigs
```

The total number of nucleotides removed by putative hemizygous deletions varied across isolates and was linearly correlated with the time elapsed since isolation (Fig. S4), apparently reflecting a systematic artifact of laboratory culturing. Thus, the extent of hemizygous deletions distinguishes the isolates and confirms that the observed genetic similarity among the L-clade isolates is not a result of culture contamination. (See also `global_thaps_clones/scripts/larrys/tic/`).

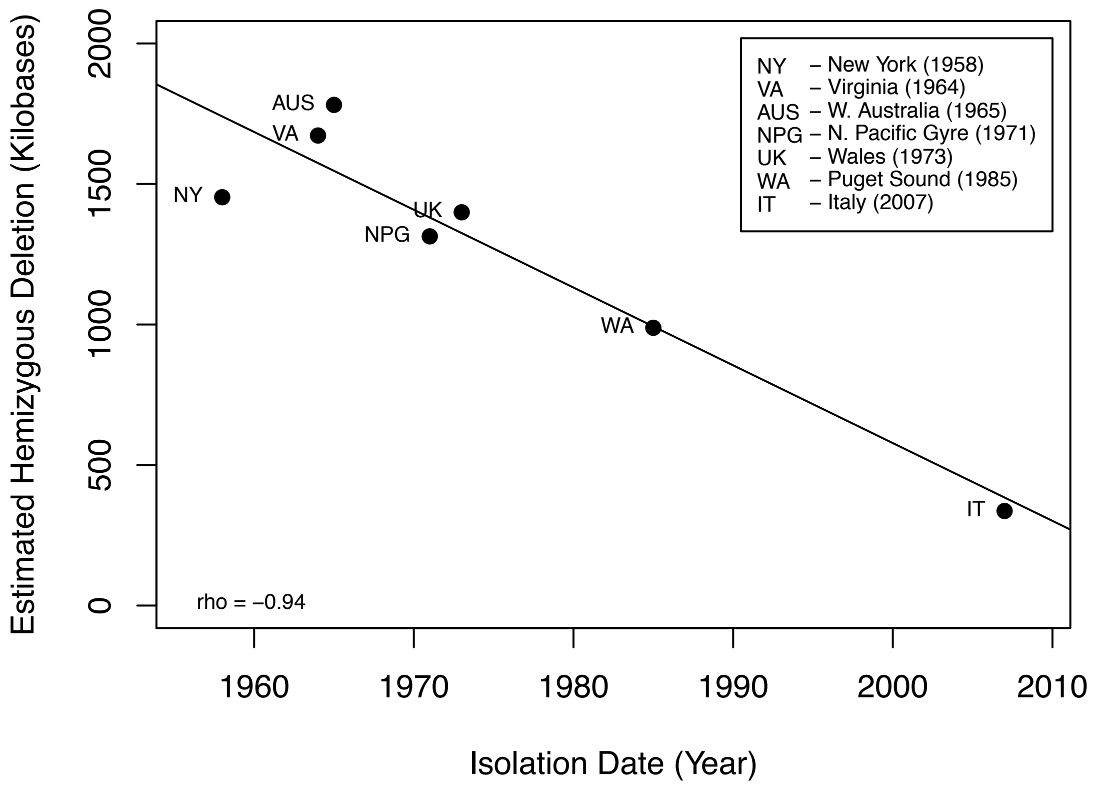

Figure S4. Extent of hemizygous chromosomal deletions in *T. pseudonana* isolates correlates with time in culture. Pearson correlation $\rho = -0.94$.

3 Refined SNP Calls

SAMtools inferred 474,613 unique variant positions among the seven isolates. It is appropriate that SNP calls should be conservative, to avoid many false positives, but, when a position was called a SNP in one strain, we often saw a significant number of reads for the same non-reference nucleotide at that position in other strains, even if they were not originally called as SNPs in these strains. We define these to be "refined" SNPs if they satisfy the criteria given below. Note that greater than 84% of all positions have no reads with a non-reference nucleotide and only a small fraction (2–3%) have 2 or more non-reference reads. Thus, identifying a position as a refined SNP based on a low count in agreement with a called SNP in another isolate should have a low false positive error rate. Refined SNPs are called by the following process:

1. Identify SNPs within each isolate using SAMtools (Methods in main text).
2. Let Δ be the union of all SNP positions across isolates.
3. For each position $\delta \in \Delta$:

- Disregard low counts: if, at position δ in a given strain, the number of aligned reads reporting a given non-reference nucleotide is < 2 in absolute count or < 5% of the total number of reads aligned there, then the count is treated as zero.
- Remove tri-allelic positions: if position δ shows nonzero counts on two or more different non-reference nucleotides across the 7 isolates, then remove δ is from Δ.

4. Position δ is a refined SNP in isolate *i* if δ is in the resulting reduced set Δ, and it shows a non-zero count in strain *i* on any of the three non-reference nucleotides. (Note that, based on the previous step, all strains declared to have a refined SNP at δ will have nonzero counts on the same non-reference nucleotide.)

For example, consider the following sets of read counts, from three positions on Chromosome 1:

**Chr**	**Pos**	**Ref**	**Isolate**	**A**	**G**	**C**	**T**	**SNP**	**rSNP**	**exon**	**indel**
Chr1	1055	G									
			1007	0	41	0	2	0	0	TRUE	FALSE
			1012	1	63	0	8	0	1	TRUE	FALSE
			1013	1	62	0	8	0	1	TRUE	FALSE
			1014	1	26	0	8	1	1	TRUE	FALSE
			1015	0	44	0	14	0	1	TRUE	FALSE
			3367	0	27	0	0	0	0	TRUE	FALSE
			1335	0	78	0	40	1	1	TRUE	FALSE
Chr1	8670	A									
			1007	19	0	0	7	0	1	TRUE	FALSE
			1012	36	0	0	12	0	1	TRUE	FALSE
			1013	44	0	0	12	0	1	TRUE	FALSE
			1014	10	0	0	7	0	1	TRUE	FALSE
			1015	24	0	0	11	1	1	TRUE	FALSE
			3367	18	0	0	0	0	0	TRUE	FALSE
			1335	27	0	0	6	0	1	TRUE	FALSE
Chr1	2013	T									
			1007	4	0	0	20	0	0	TRUE	FALSE
			1012	8	0	0	34	0	0	TRUE	FALSE
			1013	9	12	0	16	1	0	TRUE	FALSE
			1014	1	0	0	19	0	0	TRUE	FALSE
			1015	13	0	0	24	1	0	TRUE	FALSE
			3367	10	0	0	36	0	0	TRUE	FALSE
			1335	20	0	0	68	1	0	TRUE	FALSE

For Chr1:1055, all isolates have the greatest number of reads on the reference nucleotide (G). All but one also have greater than or equal to 2 reads on T, but their lower counts (perhaps combined with lower quality scores) presumably caused `SAMtools` to call this position as a SNP only in CCMP 1014 and CCMP 1335. By our rules above, this position also would be called a refined SNP in CCMP 1012, CCMP 1013, and CCMP 1015 because there are above-threshold read counts on both G and T. Note that 2 of 43 reads on T for CCMP 1007 is below the 5% threshold to be considered a refined SNP. The analysis for Chr1:8670 is similar in that `SAMtools` called a SNP in CCMP 1015 only. We identified a refined SNP in all isolates but CCMP 3367.

For Chr1:2013, all isolates have read counts on the reference nucleotide (T). All also have nonzero read counts on A, but low enough that `SAMtools` called this position a SNP in only three isolates. By our rules above that discount tri-allelic positions, this position would not be called a refined SNP in any isolate because, across the 7 isolates, there are above-threshold read counts on both A and G. (Both above-threshold counts happen to appear in the same isolate, CCMP 1013, but that is irrelevant to our

algorithm.) Cryptic repeats and other genome assembly and mapping errors could easily generate artifactual tri-allelic positions like this, hence excluding them from our analysis of shared SNPs is appropriate.

Of the 474,613 positions called as SNPs by `SAMtools`, less than 1.4% (6,496) fail to be included in the refined SNP list; Chr1:2013 is among these. The refined SNP calls (as exemplified by Chr1:1055 and Chr1:8670) appear to better reflect the actual pattern of shared diversity among the isolates. The greatest impact of the refinement protocol occurs with CCMP 1014, where low read coverage and quality resulted in the fewest `SAMtools` SNP calls. By using the low but consistent coverage, the refinement protocol identified 68% more refined SNPs in CCMP 1014 than the number called by `SAMtools`, resulting in a total that is comparable to the other L-isolates. The other six isolates gained 9%–23% SNPs (Table S1).

The percentage of SNP overlap of each isolate against the CCMP 1335 reference strain, as reported in Tables 1 and S1, is calculated as follows:

1. Calculate refined SNPs as described above.
2. For each isolate i, let σ_i be the set of refined SNPs found in i,
3. $\text{Percentage overlap} = \frac{|\sigma_i \cap \sigma_{1335}|}{|\sigma_{1335}|}$

Table S1 also shows how this overlap changes compared to the analogous statistic based on `SAMtools` SNP calls. Again, the effect is largest on CCMP 1014. The details and R code for this SNP concordance analysis are located in the `global_thaps_clones/scripts/larrys/shared-snps/` directory.

Table S1: Sequencing coverage and genomic variation among 7 Isolates of T. pseudonana before and after application of read quality filtering (Methods) and SNP refinement.

Isolate ID	Location	Coverage[1]	SNP Count[2]	SNPs Shared with 1335[3]
CCMP 1335	New York, USA	108 → 82	154K → 180K	100.0% → 100.0%
CCMP 1007	Virginia, USA	37 → 28	161K → 183K	87.5% → 92.8%
CCMP 1012	Perth, W. Australia	71 → 51	166K → 186K	89.5% → 94.3%
CCMP 1015	Washington, USA	62 → 49	175K → 190K	93.9% → 97.1%
CCMP 1014	N. Pacific Gyre	33 → 14	89K → 150K	52.1% → 78.2%
CCMP 1013	Wales, UK	70 → 45	248K → 304K	40.3% → 61.7%
CCMP 3367	Venice, Italy	64 → 45	248K → 291K	40.5% → 59.6%

[1]Mean genome sequence coverage before and after read quality filtering (see Methods, main text)
[2]Numbers of `SAMtools` SNP calls and refined SNP calls
[3]Percent of SNPs in a given isolate that are also present in CCMP 1335 before and after SNP refinement

4 Non-reference Read Statistics, R

The statistic R is defined in Methods in the main text. In the absence of error, R will be exactly 0.0 or 1.0 at homozygous positions, and ≈ 0.5 (approximate due to binomial sampling) at heterozygous sites where

the reference nucleotide is one of the two alleles. (Heterozygous sites lacking the reference nucleotide are rare.)

4.1 BWA Alignment Bias

BWA[3] has a known bias in favor of reads that agree with the reference genome used in the alignment regardless of read quality, and a bias against non-reference reads, even high-quality ones. Reads covering positions closer to a true variant (on either side) have a greater chance of also containing the variant, and thus the mapping bias causes the number of aligned reads to decrease linearly with proximity to the variant (Fig. S5).

For a diploid organism with a single copy each of two different alleles, the proportion of reads that accumulate to each allele should theoretically be 0.5 ± binomial sampling noise. The mapping bias results in R ratios (main text Methods) centering nearer to 0.4 rather than the theoretical value of 0.5 (e.g., histograms in Fig. 1 main text and S6). Additionally, this bias frequently caused BWA to map a few low-quality "reference" reads atop many high-quality "non-reference reads", thus obscuring probable homozygous non-reference positions. When calculating R, we mitigated this bias by applying additional "quality filtering" steps to the read data to remove low-quality base calls (based on color space quality scores of both di-nucleotides covering the nucleotide in question), even if they happen to match the reference sequence, as detailed in the Methods, main text.

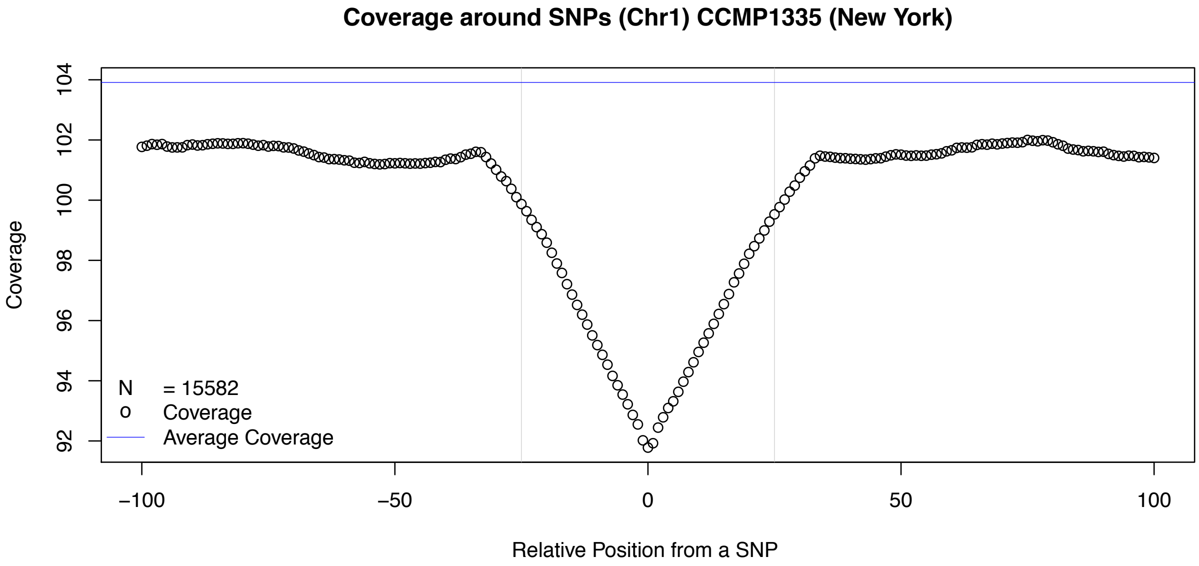

Figure S5. Read depth near SNPs reveals systematic bias against non-reference sequence. Plotted is the read depth within ±100 nucleotides of single nucleotide polymorphisms (SNPs) identified by SAMtools, averaged over all 15,582 SNPs called on Chromosome 1 of CCMP 1335. Horizontal blue line: average read depth across Chromosome 1. Vertical grey lines are at ±25bp from SNP; short read data for CCMP 1335 included a mixture of 25bp (the majority) and 35bp reads.

4.2 Non-reference Read Statistics, R, for all 7 isolates

In the presence of read- and alignment errors, R may be pulled away from the theoretically expected 0.0, 0.5, 1.0 values. The alignment bias (Section 4.1) slightly lowers the average R-value at heterozygous sites, while errors at homozygous sites can make R slightly greater than zero or slightly less than one. Nevertheless, R-values clustering near 0.0, 0.5 and 1.0 are still expected and empirically observed—in each isolate $R \approx 0.0$, $R \approx 0.5$, and $R \approx 1.0$ (in the H-isolates) are strongly favored. Additionally, the joint distribution of the R statistics for each pair of isolates is similarly clustered; e.g., for a pair of isolates (x, y) we commonly see $(R_x, R_y) \approx (0.5, 0.5)$, as would be expected for shared heterozygous positions.

These patterns are illustrated in Figures 1 and S6, where we show both the joint distribution of the R statistics for each isolate versus 1335 and the corresponding derived (marginal) distributions for each isolate alone. For visual clarity, these figures were restricted to the 35,291 positions on Chromosome 1 where (a) coverage is simultaneously between 10 and 120 reads for all seven isolates, and (b) at least one isolate has $R \geq 0.1$. Condition (a) avoids sites with unusually high or low coverage, often symptomatic of problems with the reference sequence such as collapsed repeats. Condition (b) avoids the great preponderance of sites where the reference nucleotide is homozygous in all seven strains ($R \approx 0.0$), thus highlighting the more interesting situations where genetic diversity is present.

Six isolates cleanly partition into two groups: the L-clade members share most of their heterozygous positions (dense cloud near (0.5,0.5) in the pairwise plots) and have few apparent homozygous non-reference positions (all L-isolates have fewer than 16K positions with $R > 0.8$, fewer than ~10K after subtracting apparently hemizygous regions (Section 2.3)). In contrast, the two H-isolates each have > 100K apparently homozygous non-reference positions. Furthermore, the many heterozygous and homozygous reference positions are shared randomly (as expected in HWE). CCMP 1014 is a partial outlier in these analyses, which we attribute to low coverage/low quality in that sequencing run, but it does exhibit the L-clade characteristics of having few positions with $R \approx 1$, and many heterozygous positions shared with the other L-clade members, as evidenced by the cloud of points around (0.5,0.5) in the comparison to CCMP 1335, and SNP "deserts" that largely overlap those found in other L-clade members.

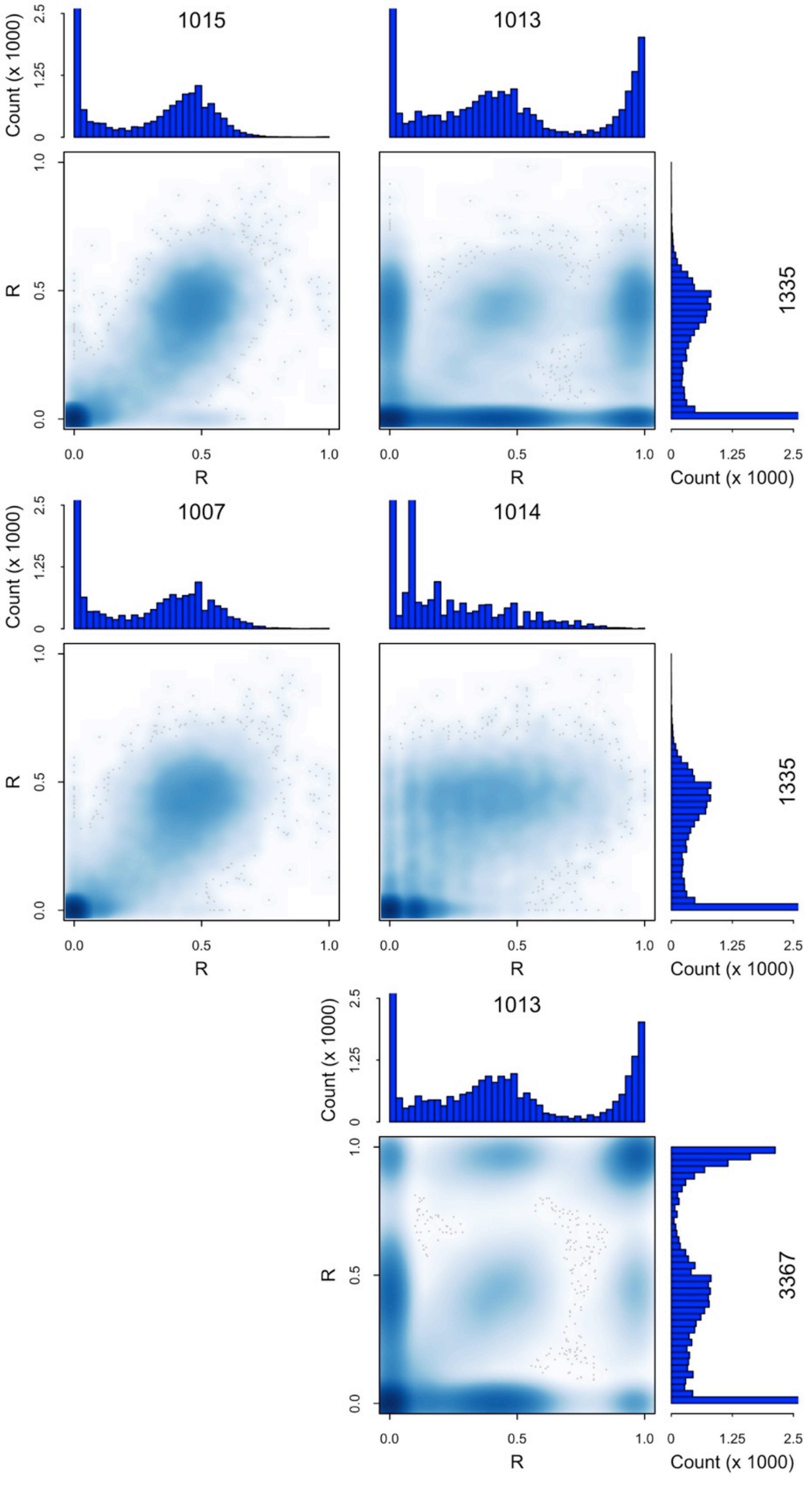

Figure S6. Per position non-reference coverage fraction, R, based on comparisons between CCMP 1335 and the 4 other L-isolates (top and middle panels), as well as the comparison between the H-isolates (bottom panel) CCMP 1013 and CCMP 3367 at a shared set of ≈ 35K positions. Read counts are ×1000. See Fig. 1 caption and Supp. Section 4 for details on figure annotations.

5 Hardy-Weinberg Equilibrium, Sampling, and Recombination

5.1 The Reference Genome is a "Hardy-Weinberg Haplotype"

We examined how often the reference sequence reflects rare alleles at polymorphic sites. When a haploid "reference genome" is constructed from a single diploid individual, homozygous positions are recorded in the reference; for heterozygous positions, one of the two alleles is selected to be the reference nucleotide essentially at random, e.g., based on which variant accumulated more reads in the sequencing run. The construction of the reference sequence can be cast as an explicitly probabilistic process, assuming a population in Hardy-Weinberg Equilibrium (HWE) and ignoring read errors and potentially biased coverage. Draw two samples from the HWE population at each genomic site; if the two samples differ, choosing the one with more reads is equivalent to choosing the first draw, since they are equi-probable; if the two samples agree, it is again equivalent to choosing the first draw. So, the reference sequence is equivalent to drawing one sample from HWE at each position. I.e., the reference genome is a "Hardy-Weinberg haplotype"—equivalent to a randomly selected haplotype drawn from the HWE population.

The CCMP 1335 reference sequence[1] was derived from an isogenic cell culture: the sequencing project isolated a single cell from the CCMP 1335 strain, then cultured it to produce DNA for sequencing. Reproduction in culture is believed to have been exclusively mitotic, and so the reference construction model presented above is as appropriate as it would be for a sequencing project based on a sample from a single multicellular organism. Unobserved sexual reproduction in culture might increase the variance in observed read counts at heterozygous sites, but with rare exceptions (e.g., homozygous lethality), should not alter the mean 50-50 mixture of the two alleles.

5.2 Heterozygous Sites Outnumber Homozygous Non-Reference Sites 2 to 1

In both H-isolates, we see a roughly 2:1 ratio between numbers of heterozygous and homozygous non-reference positions (e.g., Fig. S7). In principle, that ratio depends on both the distribution of allele frequencies in the sampled populations and on the reference genome. For example, the 2:1 ratio is predicted by HWE if all non-reference alleles have a frequency of 0.5. However, the neutral theory of molecular evolution predicts that most allelic variation is rare. Therefore, within a re-sequenced genome, homozygous non-reference positions should be rare if the reference genome exclusively records major alleles (those with highest frequency, typically ≥ 0.5) at polymorphic sites.

But in fact, the 2:1 ratio *is* expected, *independent* of allele frequencies, given the way the reference genome was constructed. Specifically, the reference sequence is effectively a random haploid genome (Section 5.1) assumed to reflect population-level allelic frequencies across the length of the genome, where major alleles are recorded at most polymorphic positions, but rare alleles are recorded with proportionally rare frequencies. In re-sequenced individuals, homozygous non-reference positions will be rare at those positions where the reference sequence records the major allele, but they will be common at the positions where the reference sequence has captured rare alleles. These effects counterbalance to yield the observed 2:1 ratio—*exactly* 2:1 is expected when only bi-allelic positions are considered, and (slightly) greater than 2:1 when (typically rarer) multi-allelic positions are considered, as shown below.

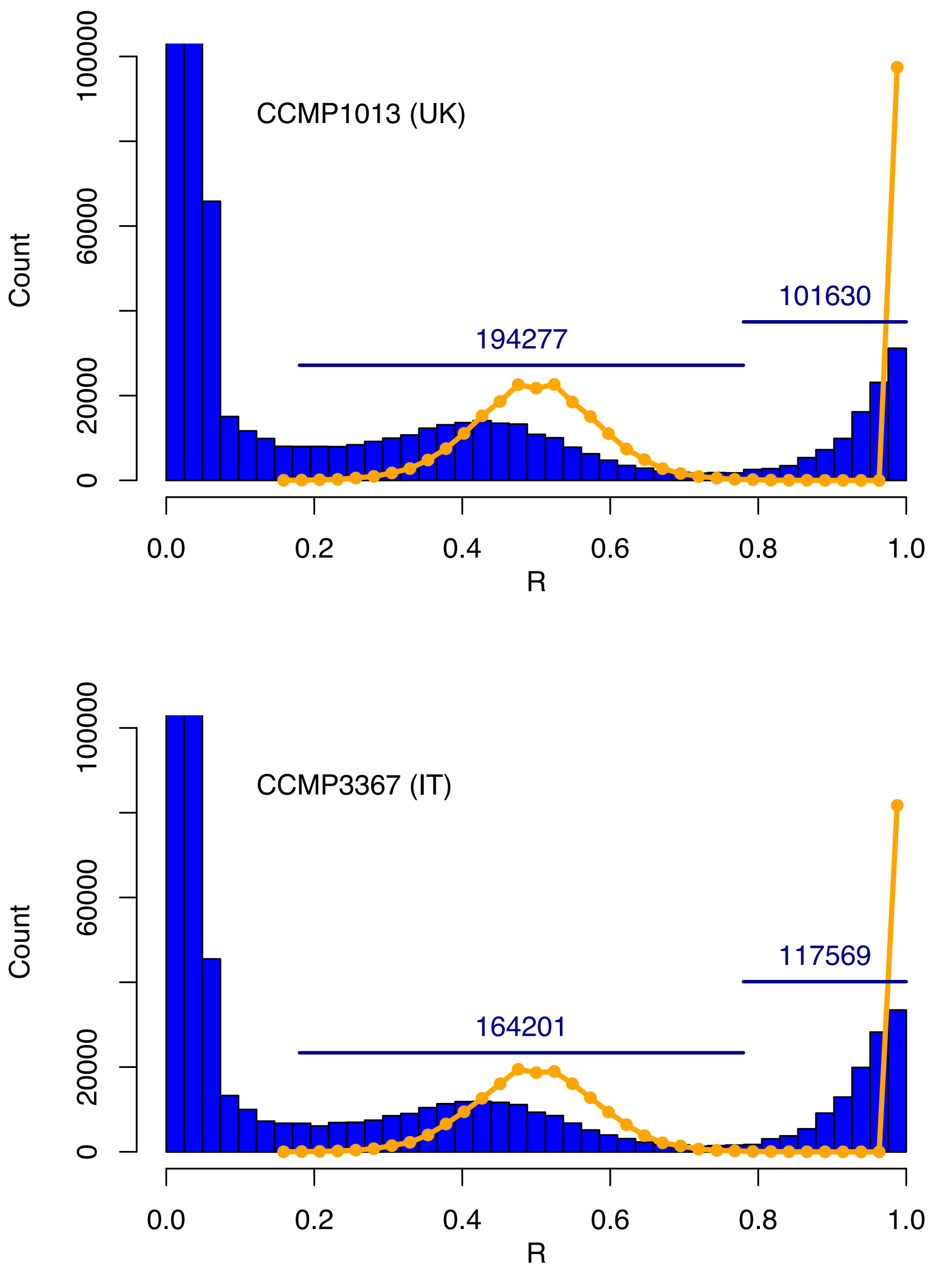

Figure S7. Numbers of heterozygous and homozygous non-reference positions exhibit Hardy-Weinberg proportions in the H-isolates CCMP 1013 (top) and CCMP 3367 (bottom). Histograms of R statistics (blue bars, truncated at count = 100K) are compared to the theoretical Hardy-Weinberg R-distributions (orange curve). Numbers above horizontal lines are the numbers of positions with $0.18 \leq R \leq 0.78$ (left line) and $0.78 < R \leq 1$ (right line) and represent estimates for potential heterozygous and homozygous non-reference positions, respectively. The ratio between the numbers of heterozygous and homozygous non-reference sites approximates the theoretically predicted 2:1 ratio expected for isolates sampled from a wild population in Hardy-Weinberg Equilibrium. Histograms reflect all chromosomal positions having coverage within 1 standard deviation of the isolate-specific mean, ≈ 27 million positions in each case.

For example, consider 100 bi-allelic loci, each with a 0.1 minor allele frequency in a population in HWE. At each locus, $0.9^2 = 81\%$ of individuals are expected to be homozygous for the major allele, $2 \cdot 0.9 \cdot 0.1 = 18\%$ heterozygous, and only $0.1^2 = 1\%$ homozygous for the minor allele. In any given individual, the number of homozygous non-reference positions depends on the reference. If the reference reflects the major allele at each locus, then 1% of these loci will be homozygous non-reference (vs 18% heterozygous, an 18:1 ratio), but if the reference records the minor allele at 10% of loci (as expected in a random haplotype from this population), then the number of homozygous non-reference loci is expected to be 0.81 * 10 + 0.01 * 90 = 9.0, so the heterozygous:homozygous non-reference ratio is 2:1.

More generally, consider a diploid population in Hardy-Weinberg equilibrium. Focus on a specific bi-allelic position having minor allele frequency $0 \leq q \leq 1/2$ and $p = 1 - q$. When re-sequencing another individual drawn from the same population, determining whether this position is heterozygous versus homozygous non-reference can be visualized as drawing three independent samples from the HWE population—the first draw determines the reference haplotype, and the other two define the genotype of the new individual. If all three are the same, that site is homozygous for the reference allele. If the three are not all the same, then only three distinct possibilities are relevant: Letting "a" denote the allele that was observed only once, and "b" the allele seen twice, the three draws yield abb, bab, or bba. Since the first letter defines the reference, outcome abb is the homozygous non-reference case, and the other two outcomes are heterozygous. These three outcomes are equally likely (with all three probabilities equal to p^2q or all equal to q^2p, depending on whether "b" is the major or minor allele, respectively), so the heterozygous to homozygous non-reference ratio is 2:1. Inclusion of (a small number of) 3- and 4-state positions in the population will raise the proportion of heterozygous positions in a re-sequenced individual (by a similarly small amount).

5.3 CCMP Re-Sequencing Cultures Are Isogenic

The CCMP 1335 reference sequence[1] was derived from an isogenic culture that originated with a single isolated cell of *Thalassiosira pseudonana*, strain CCMP 1335. In contrast, the cultures of each "re-sequenced isolate" were grown from ~5–10 cells isolated by flow cytometry from each CCMP strain. Genetic diversity in the re-sequencing culture could potentially mask genomic signals of interest. For example, a site that is homozygous non-reference in some but not all cells might be indistinguishable from a uniformly heterozygous site. A priori, it is plausible that all seven CCMP cultures are isogenic, but to be conservative, we looked to our data for direct confirmation.

Suppose one of the CCMP cultures had several founder cells (f) that were independently drawn from an HWE population. Extending the analysis from Section 5.2, at a bi-allelic position having minor allele frequency $q = 1 - p \leq p$, the probability that the $2f$ chromosomes of the f founder cells hold exactly $j = 0, \ldots, 2f$ copies of the non-reference allele is:

$$B(j,f) = p\binom{2f}{j}p^{2f-j}q^{j} + q\binom{2f}{j}p^{j}a^{2f-j}$$

This is the probability of exactly j "successes" when performing $2f$ trials in a weighted mixture of two binomial distributions, one with weight p and success probability q, and the other with weight q and success probability p. Graphically, the probability mass function for this system will place all mass at the discrete points $j/(2f)$, j = 0, . . . , $2f$ (Fig. S8).

According to neutral theory, we should expect many positions to exhibit small minor allele frequencies q. Intuitively, when q is sufficiently small, the most likely scenario is that the major allele is the reference

nucleotide. In this case, the most likely number of copies of the non-reference allele captured among the f founders is $j = 0$, with $j = 1$ being next most likely, and $j = 2, 3, \ldots$ being increasingly unlikely (the first term in the formula above). However, if the minor allele is the reference nucleotide (which happens with probability q), then the most likely outcome is that $j = 2f$ non-reference alleles (i.e., only major alleles) are seen, with $j = 2f - 1, 2f - 2, \ldots$ being increasingly unlikely (the second term in the formula). These two series cross at $j \approx f$, and their sum is minimized when $j = f + 1$, with the net result that $B(j, f)$, as a function of j, is convex ("U-shaped"), with its minimum near the middle, and peaks at the extremes j=0 and j=2f. The only exception is when $f = 1$ (when the important $j = 1$ case is the middle). This holds for any single site with $q \leq 1/(4f + 1)$, and, since sums of convex functions are themselves convex, for any mixture of sites with minor allele frequencies at or below $1/(4f + 1)$.

The R distributions (analogous to those in the histograms of Figs. 1, S6, and S7) expected from the model outlined here would reflect (a) a theoretical distribution similar to the dots shown in Fig S8 for alleles captured in the founder population, but (b) summed over many positions with varying minor allele frequencies, and (c) "blurred" by stochastic sampling as the sequencer accumulates reads from both alleles at heterozygous sites. The gray bar graphs in Fig S8 reflect a simple simulation of this (coverage of 48 and binomial sampling of both alleles equally at all sites, with no errors or bias in sequencing or mapping). Note that the "U" shaped scenario does not apply individually to larger q values (red and green points in the bottom panels of Fig S8), but does apply collectively to a mixture including many sites with small q, even when some sites with larger q are present, as shown in that figure. In aggregate, these effects add variability to the data, but do not alter the main features of our model, namely, presence of a fair number of positions with apparent non-reference frequency near 1.0, and, with one key exception, absence of a peak in the R-distribution near 0.5. The key exception is when f is one: establishment of the culture from a single cell (or, equivalently, 5–10 genetically identical cells) means that all heterozygous sites are retained at a 50-50 allele frequency in the descendant population (as fixed heterozygous sites in all offspring if only mitotic division happens in culture, and maintained on average if there is unobserved sexual reproduction in culture).

Thus, the presence of the peak near 0.5 in the R histograms (e.g., Figures 1 & S6) for 6 isolates demonstrates that these re-sequencing cultures were isogenic (having been established from, or eventually dominated by the descendants of, a single isolated cell). An important consequence is that the exact number of cells used to establish the re-sequencing culture (the 5–10 cell estimate) is not relevant for our subsequent analysis—all are genetically identical. (Interpretation of CCMP 1014 is hampered by lower data quality, but confirmation of the a priori expectation of isogenicity in the 6 other isolates supports the hypothesis that 1014 is also isogenic.)

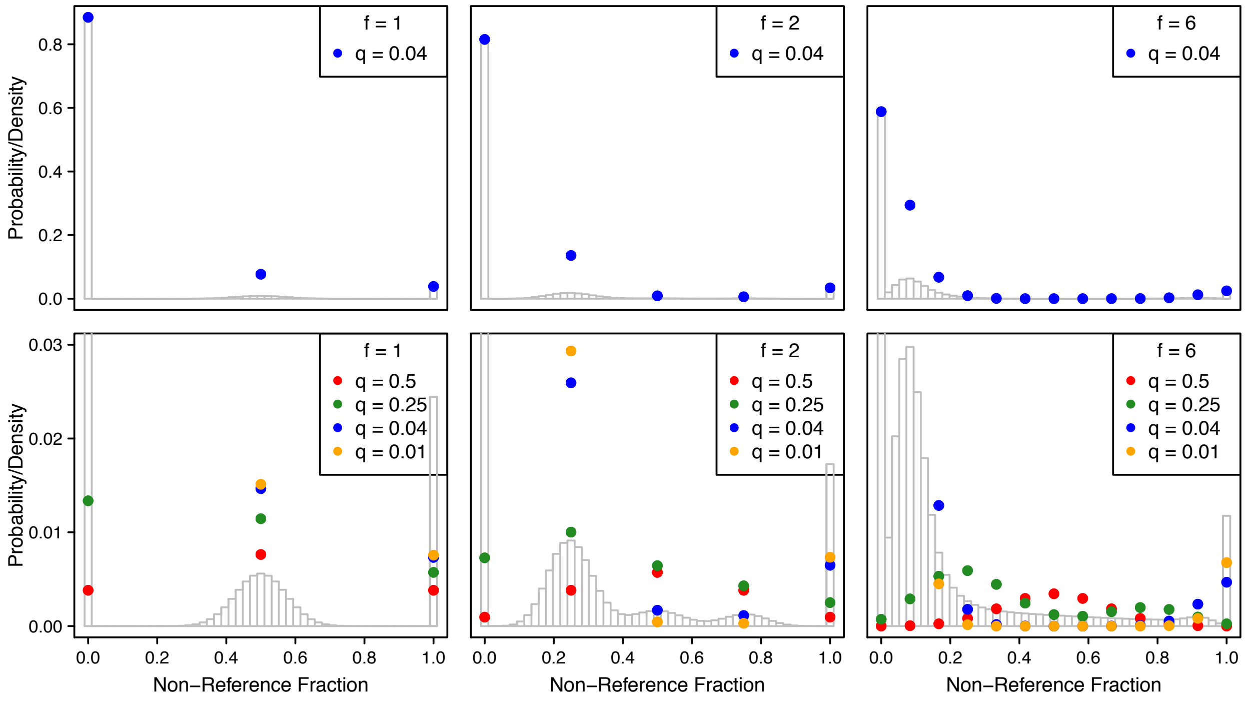

Figure S8. Modeling distributions of non-reference sequence reads. Top row: theoretical and simulated results assuming that re-sequenced cultures were based on $f = 1$ (left), 2 (middle), or 6 (right) founder cells sampled from a HWE population. At sites having a minor allele frequency of $q = 0.04$, the probability (y-axis) that these f cells hold a specified fraction $j/(2f)$, $j = 0, \ldots, 2f$ (x-axis) of the non-reference allele at that site is plotted (blue dots). The superimposed gray bar graphs simulate the effect of stochastic sampling of reads during sequencing—e.g., in the upper left panel, 8% of bi-allelic sites having this minor allele frequency would be expected to be heterozygous (blue dot at $x = 0.5$), but sampling of reads for both alleles will spread their apparent non-reference proportions as shown ("bump" in the gray bar graph visible from $x = 0.4$ to 0.6, roughly). The simulation assumes a coverage of 48 at all sites, with no errors or bias in sequencing or mapping. Bottom row: analogous graphs, assuming a weighted mixture of minor allele frequencies q (see legend), with weights inversely proportional to q. (Note the change in y scale in the lower row; the leftmost points and gray bars are clipped to expose more detail at small y values).

5.4 Estimating Crossover Density

To independently corroborate the lack of meiosis along the lineages joining the L-isolates, we estimated the density of crossover events between each pair of isolates using the `Genome Analysis Toolkit`[4] (`GATK` v3.3.0) as well as the command line tools of the `Picard` software package (v1.119). `BAM` files (Methods of main text) were used in the following analyses.

• Preprocessing `BAM` files: Thaps3.all.fasta contains version 3 of the CCMP 1335 reference genome, each `aln.bam` holds aligned reads from one isolate and `alnRG_sorted.bam` is the resulting sorted `BAM` file.

```
java -jar AddOrReplaceReadGroups.jar INPUT=aln.bam OUTPUT=alnRG.bam \
     RGID=group1 RGLB=lib1 RGPL=illumina RGPU=unit1 RGSM=sample1
```

```
java -jar  SortSam.jar INPUT=alnRG.bam OUTPUT=alnRG_sorted.bam \
      SORT_ORDER=coordinate
java -jar BuildBamIndex.jar INPUT=alnRG_sorted.bam
```

- Create a `GATK VCF` file (the variant calls, `output.vcf`):

```
java -jar GenomeAnalysisTK.jar -T HaplotypeCaller \
      -R Thaps3.all.fasta –I alnRG_sorted.bam -o output.vcf
```

- Physical Phasing:

```
java -Xmx2g -jar GenomeAnalysisTK.jar -R Thaps3.all.fasta \
      -T VariantFiltration -o output_filtered.vcf --variant output.vcf\
      --filterExpression "MQ > 40 && QD > 6" --filterName "PASS"
java -jar GenomeAnalysisTK.jar -T ReadBackedPhasing \
      -R Thaps3.all.fasta  -I alnRG_sorted.bam \
      --variant output_filtered.vcf -o output_phased.vcf
```

Physical phasing, when successful for a given pair of SNP positions, shows which pairs of variants lie on the same chromosomes. For example, given a G/T SNP at one position and an A/G SNP at another position on the same homologous pair of chromosomes, phasing attempts to determine whether G—A appear together on one chromosome and T—G on the other, versus G—G on one and T—A on the other. Using the physical phasing data, the density of crossover events between two strains was estimated as follows:

1. Generate a list of pairs of SNPs that are phased in both strains. Each pair defines a genomic interval.
2. Select as many pairs as possible subject to the constraint that their genomic intervals do not overlap (algorithmically, an instance of the Interval Scheduling Problem[5])
3. Count the number of observed crossover events by examining whether a pair of SNPs is phased differently between the two strains.
4. Estimate the actual number of crossover events from the observed counts by simulation.
5. Calculate the estimated crossover density by dividing the estimate from the previous steps by the total length of the set of pairs examined.

Physical phasing with our sequencing data does not recover two complete haplotypes per chromosome. Rather, we find many small "patches," often interleaved, where multiple reads or mate pairs covering the same two heterozygous positions consistently show the same allele association, and hence can be phased. When such a pair is observed to phase differently between two strains, we infer that an odd number of crossovers have occurred between them. Removing overlaps (step 2) prevents double counting of observed events, while extracting a maximal set of non-overlapping pairs reduces their average separation, thus reducing the chance of multiple crossovers between a pair.

Table S2A shows the density of "observed crossovers" (number of events per kilobase) identified in step 3 of the above procedure. Implicitly, this procedure treats any odd number of crossovers in an interval as one, and any even number as zero, which causes the observed crossover count to underestimate the true count. The simulation in step 4 corrects for this as follows: place a given number x of simulated crossovers at random in the intervals selected in step 2, then count the number of them that would be observed in those intervals (i.e., count intervals containing an odd number of simulated crossovers). Repeat with different values of x and different random placements until 20 trials yield simulated counts that are within some small tolerance of the actual count observed in step 3. Step 4 reports the average of those 20 "x" values, and the resulting estimated densities are shown in Table S2B. The median length of

phased intervals is comparable to the insert length in the mate paired sequencing data (≈2.5 Kb). Given that, and observed crossover densities ≤ 0.07 per Kb in all L:L comparisons, double-crossovers are rare and the adjustment described above has a modest effect. Given the sharply higher baseline of observed density in all H:L and H:H comparisons (Table S2A), the adjustment is much more substantial there (Table S2B).

Table S2. Crossover densities (per kilobase) between each pair of isolates. Bold: L-isolates; Red: values greater than 0.2. Panel A gives observed densities. Only an odd number of crossovers within a haplotype block are observable. Panel B gives estimated densities after adjusting for unobserved events.

A. Observed Crossover Densities						
	**1007**	**1012**	1013	**1014**	**1015**	3367
**1335**	0.030	0.036	0.236	0.070	0.037	0.269
**1007**		0.039	0.209	0.051	0.037	0.235
**1012**			0.204	0.049	0.057	0.207
1013				0.254	0.210	0.214
**1014**					0.052	0.304
**1015**						0.215

B. Adjusted Crossover Densities						
	**1007**	**1012**	1013	**1014**	**1015**	3367
**1335**	0.032	0.040	2.065	0.086	0.040	2.838
**1007**		0.043	1.260	0.058	0.041	2.330
**1012**			1.028	0.056	0.066	1.713
1013				0.840	1.031	1.338
**1014**					0.060	1.703
**1015**						1.757

The crossover density presented in Table 1 (main text) reports these results for the comparison of the reference CCMP 1335 strain to each of the others, i.e., the first row of Table S2B.

We have argued that the L-isolates are related exclusively mitotically. Hence, crossovers in the L:L comparisons are unexpected. We speculate that many of them are "false positive" artifacts induced by genome assembly, sequencing, and phasing errors. We are unable to estimate the magnitude of such effects, but we have no reason to expect that they explain the large gap between estimated L:L crossover densities and H:L or H:H densities; if anything, we expect such errors to have inflated the L:L density estimates more. In short, we are confident that the crossover densities in all comparisons to an H-isolate are significantly greater than all L to L comparisons.

See the iPython notebooks in `global_thaps_clones/scripts/Nao/` for details of this analysis.

5.5 The H-clade Retains Sexual Reproduction but L-clade Isolates are Mitotic Descendants of a Common Ancestor

We see no characteristics of the two H-isolates that are at odds with Hardy-Weinberg Equilibrium or other characteristics expected of normal, sexually reproducing eukaryotes. On the other hand, the five L-isolates sharply diverge from these expectations. Specifically, they are clonal: they appear to reflect purely mitotic descent from a common ancestor. This conclusion is based on several observations. First is the conclusion that each CCMP isolate arose from the equivalent of a single founder cell (see Section 5.3). Second, assuming that each isolate was drawn from a common population in HWE, each (non-1335) founder cell would have a heterozygous to homozygous non-reference ratio of at most 2:1 with respect to the CCMP 1335 reference, as shown in Section 5.2. Homozygous non-reference positions in the founder will appear exclusively non-reference in its descendants, even if recombination were occurring in culture,

for the simple reason that no alternative nucleotide exists at that position in any cell in the culture. In consequence, the 2:1 ratio will be recapitulated when re-sequenced. Thus, the few homozygous non-reference positions (≤ 10091 positions with R ≥ 0.75) observed in the L-isolates relative to the ≈90K predicted by this analysis (1/2 of the ≈180K observed heterozygous positions reported in Table 1) argue strongly against even a single meiosis anywhere along the lineages joining the five L-isolates.

Additionally, in HWE, sites that are polymorphic in the population will, of course, randomly assort into all possible heterozygous and homozygous states in individuals. Thus, the high degree of concordance of heterozygous positions across the L-clade is unexpected and provides the basis for a quantitative test of adherence to the HWE model. Specifically, assume that the L-isolates comprise 5 independent samples from a population in Hardy-Weinberg equilibrium. Designate one isolate, *A*, as a "template." Let the (unknown) allele frequencies at a randomly chosen heterozygous position (SNP) within the template be p_1 and $q_1 = 1 - p_1$. (Positions having 3 or 4 nucleotide variants segregating in the population are assumed to be negligibly rare.) Under HWE, a second isolate *B* will also be heterozygous at the same position with probability $2p_1q_1 \leq 1/2$. The probability that this position is heterozygous in a third isolate, *C,* is also $2p_1q_1 \leq ½$, *independently* and the same is true for isolates D and E. Consequently, the probability that a heterozygous position in *A* is simultaneously heterozygous in the other 4 isolates (a "concordant" position) is at most $1/2^4 = 1/16 = 6.25\%$. In contrast, choosing CCMP 1014 as the template, we find 78% of its 89184 heterozygous positions are concordant across the L-isolates. Unrefined SNPs called by `SAMtools` are used because their determination is independent of SNP calls in other isolates, unlike refined SNPs whose identification depends on SNPs in other isolates (Section 3). A 78 % concordance of SNPs is astronomically unlikely if they are unlinked (as assumed under HWE). Recognizing that linkage disequilibrium is possible in wild populations, we conservatively posit a second model ("partial HWE") where all SNPS on one chromosome are linked, but SNPs on different chromosomes segregate independently. Under this assumption, a second heterozygous position chosen at random in *A*, *on a different chromosome* (to avoid linkage to the first SNP) with allele frequencies, say, p_2 and $q_2 = 1 - p_2$, will be a SNP in *B, C, D* and *E* with a probability of $(2p_2q_2)^4 \leq 1/16$, independently of position 1. Repeating this for the 24 chromosomes in *T. pseudonana*, the number of five-way concordant positions observed should be dominated by the number observed when sampling from a binomial distribution with parameters $n = 24$ and $p = 1/16$. This "partial HWE" distribution has a mean value of at most 24/16 = 1.5 concordant positions in samples of size 24, whereas, as noted above, the observed distribution has a much higher mean of 24*78% > 18.7. The probability of observing 18 or more unlinked concordant positions in a sample of 24 under the "partial HWE" null model is less than 2×10^{-17}. A random sets of unlinked SNPs sampled from the template will not always yield 18 concordant ones, of course, and sets with fewer concordant ones are more probable under the partial HWE model, but averaging over possible sets of 24 positions still yields a probability of less than 7×10^{-10} under the partial HWE model of seeing data as concordant as we observe. This was calculated (using R) as follows.

First, the probability that we would observe $0 \leq i \leq 24$ concordant positions in a sample of 24, given that 78.39% of positions are concordant follows this binomial distribution:

```
x.equals.i.distribution <- dbinom(0:24, 24, fil.fiveway.percent/100)
print(x.equals.i.distribution, digits=3)

# [1] 1.07e-16 9.33e-15 3.89e-13 1.04e-11 1.97e-10 2.86e-09 3.29e-08 3.07e-07 2.37e-06 1.53e-05
#[11] 8.31e-05 3.84e-04 1.51e-03 5.05e-03 1.44e-02 3.48e-02 7.11e-02 1.21e-01 1.71e-01 1.96e-01
#[21] 1.78e-01 1.23e-01 6.09e-02 1.92e-02 2.90e-03
```

Second, the p-value (assuming partial HWE) corresponding to $0 \leq i \leq 24$ observed concordant positions follows a different binomial distribution:

```
p.val.of.x.equals.i <- c(1, pbinom(0:23, 24, 1/16, lower.tail = F))
print(p.val.of.x.equals.i, digits=3)

#  [1] 1.00e+00 7.88e-01 4.48e-01 1.87e-01 5.95e-02 1.49e-02 3.01e-03 4.99e-04 6.90e-05 8.02e-06
# [11] 7.89e-07 6.60e-08 4.72e-09 2.87e-10 1.49e-11 6.59e-13 2.46e-14 7.66e-16 1.98e-17 4.14e-19
# [21] 6.88e-21 8.70e-23 7.88e-25 4.56e-27 1.26e-29
```

The key point is that most sets of 24 unlinked SNPs will contain many 5-way concordant positions, since 78% of all positions are observed to be concordant, whereas the expected number of such positions, based on the partial HWE assumption, is at most 24/16 = 1.5. To summarize these two distributions in a single number, the expected p-value based on a 24 SNP sample is the average of the latter values weighted by the former:

```
e.of.p.of.x <- sum(x.equals.i.distribution * p.val.of.x.equals.i)
e.of.p.of.x

# [1]   6.939136e-10
```

In short, it is highly improbable that 5 isolates from a sexually reproducing population in HWE, even "partial HWE" (as defined above), should share as many heterozygous positions as we see. Again, we note that even one meiosis and subsequent fertilization (even selfing) along the lineage joining two of the L-isolates would dramatically reduce the frequency of shared SNPs. We therefore conclude that the L-clade isolates reflect purely mitotic ancestry. (Note that this is not sufficient to conclude that the L-isolates are obligate asexuals. We address that question separately in Section S8.)

In contrast, the ≈2:1 heterozygous to homozygous non-reference ratio observed in the H-isolates (with respect to the CCMP 1335-based reference) is consistent with HWE (Fig. S7) and with sexual reproduction in the wild within these populations. This conclusion assumes that allele frequencies in the H-isolates have not changed drastically in the time since the L-clade founder emerged from the population that was the common ancestor to all isolates.

6 Estimating Regions of Significantly Low SNP Density

Our approach for identifying regions of unusually low SNP density ("SNP deserts") is outlined in Methods, main text. We have encoded this test into the R function `snpModel` located in `global_thaps_clones/R/allFunctions.R`. The identified loci are listed in the R-data file `global_thaps_clones/data/des.rda`. (Although not explored in this work, the method similarly identifies "SNP hotspots," i.e., regions with significantly elevated SNP densities, which are listed in `global_thaps_clones/data/hs.rda`.)

The size distributions for each isolate's deserts are shown in Fig. S9.

The blue regions in Fig. 2A (main text) show all deserts called on Chromosome 1 in all seven strains. Desert calls may include regions where the reference nucleotide is unknown (e.g., the gold region in Fig. 2A) or where there has been a hemizygous or full deletion in one of the isolates (c.f., Fig. S4). If ignored, these features might cause SNP densities to be underestimated. Hence, SNP density estimates shown in Fig. 2B (main text) were made after masking regions with deletions and unknown nucleotides. The estimated SNP density in each region is the number of unmasked SNPs divided by the number of

unmasked nucleotides in it. Estimated densities in the "intervening" regions between large deserts include (unmasked portions of) shorter deserts that may happen to fall between the large ones. For example, two shorter deserts account for more than two thirds of the 42Kb region between the 27th and 28th large deserts, thus contributing to its low apparent SNP density. (Indeed, the three non-desert regions that separate these four deserts have read coverage that is at least double the genome-wide average, suggesting that "SNPs" called there are an artifact of collapsed repeats in the genome assembly or read mapping errors. That is, a single loss of heterozygosity event likely affected this entire 200Kb section of Chr 22.)

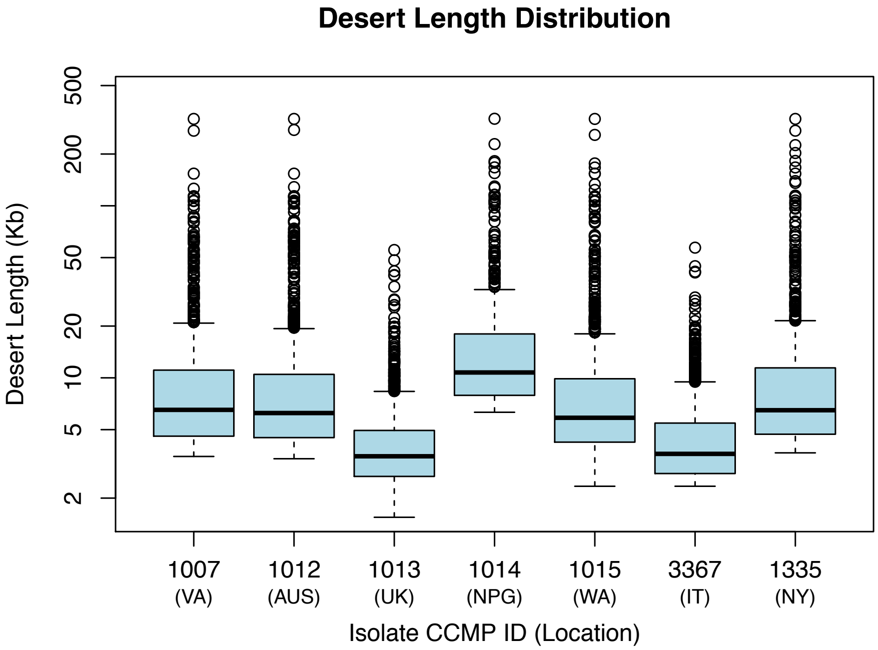

Figure S9. Genome-wide distributions of SNP desert size in *Thalassiosira pseudonana*. Tukey box-plot for the size distribution of SNP deserts for all 7 isolates of *T. pseudonana*. Y-axis shows desert lengths (in kilobases) while the x-axis indexes the isolates. Bold line = median; rectangles = inter-quartile range (IQR); whiskers = ± 1.5 upper/lower IQR; dots = outliers.

Surprisingly, many of the SNP deserts have concordant boundaries across the L-clade. For example, Table S3 illustrates three deserts that are present in all five L-isolates but not in the H-isolates. Excluding CCMP 1014, the boundaries differ among isolates by at most ±225 base pairs. The wider boundaries in CCMP 1014 are likely a technical rather than biological effect, due to lower coverage and read quality.

Table S3 Genomic coordinates of the boundaries of three of the largest SNP deserts in the L-isolates.

ID	Chromosome 1 (320 Kb[a])	Chromosome 5 (150 Kb[a])	Chromosome 5 (107 Kb[a])
CCMP 1335	1376223 : 1696217	798329 : 952251	2119568 : 2226980
CCMP 1007	1376212 : 1696217	798373 : 952251	2119647 : 2226755
CCMP 1012	1376223 : 1696402	798423 : 952251	2119647 : 2226755
CCMP 1015	1376223 : 1696217	798373 : 952251	2119647 : 2226755
CCMP 1014	1375768 : 1696402	798283 : 953293	2119193 : 2227706

[a]approximate length of desert

7 Genes Associated with Sexual Reproduction

The number of annotated genes was determined by searching the genome (JGI) for keywords flagell*, sex*, and meio* individually. The nucleotides contributing to SNPs were identified for two sexually induced genes (*SIG 1* and *SIG 2*; protein Ids 12821 and 7122, respectively) and a gene encoding a putative flagellar ribbon protein (protein Id 1495) that would be associated with sperm in diatoms. *SIG1* and the flagellar ribbon gene are both found within the 320 Kb desert on Chromosome 1. We delineated the genes from the start to stop codons using genomic coordinates for the version 3.0 gene models of the CCMP 1335 reference sequence (JGI). SNPs (based on `SAMTools` SNP calls) were counted separately in introns and exons (UTRs were excluded) using custom R scripts (`global_thaps_clones/scripts/SexGenes/`). Synonymous and non-synonymous substitutions were counted in translated protein sequences based on the alternative nucleotides at each SNP position relative to the reference sequence; reverse complements of the genomic sequence were translated where appropriate. These three genes contain no SNPs in the L-isolates. SNP counts for the H-isolates are provided in Table S4. The H-isolates do not share any SNPs in *SIG1* or *SIG2,* but they do share 7 SNPs in the flagellar ribbon protein.

Table S4: Number of H-isolate SNPs in three genes associated with sexual reproduction.

Gene	Gene ID	Chr	Start	Stop	Strand	Isolate	Intron	Exon	Syn[a]	Non-Syn[a]
SIG 1	12821	1	1537890	1540417	+	1013	0	3	1	2
						3376	1	8	5	3
SIG 2	7122	7	1338802	1340055	-	1013	0	1	0	1
						3376	1	11	9	2
(ribbon	1495	1	1667601	1668698	-	1013	-	15	5	10
domain)						3376	-	14	5	9

[a]number of synonymous (Syn) and non-synonymous (Non-Syn) changes in translated protein sequence.

It is natural to interpret the SNPS in the H-isolates has a sign of "decay" expected in a nonfunctional gene, but we find nothing conclusive, nor is that surprising. First, assuming any of these genes is causally involved, recall that we hypothesize that the SNP-free reference sequence found throughout the L-clade is the nonfunctional allele. We do not expect significant divergence between the

two copies of these (or any) genes in the LoH regions of the L-isolates in the few hundred years since the presumed LoH event that united them. I.e., the fact that the two copies of these genes are identical merely reflects the youth of this genotype, not the action of strong negative selection. Degeneration of the nonfunctional allele during the pre-LoH period of its coexistence with the functional alleles is expected, but we have no estimate for the duration of that period. If it was long enough for a nonfunctional allele to accumulate extensive damage (many nonsense mutations, reading frame shifts, splice site erosion, ...) then it is likely that the gene would not have been recognized during genome annotation (performed on the CCMP 1335 reference sequence). However, when rare, the null allele is essentially neutral, and neutral alleles are much more likely to be purged by genetic drift than to be maintained for long time spans, making it more likely that the null allele is "young" and hence less degraded, which is consistent with the moderate number of differences between the L- and H-clade genes shown in Table S4.

8 Obligate Asexuality

We have found the L-genotype at 5 of 7 sampled locations. It is reasonable to infer that the L-genotype constitutes at least a significant minority of the population at these 5 locales, otherwise it is unlikely that it would have been sampled 5 times (even if it happens to acclimate to culture conditions readily). Furthermore, as argued above, the genetic similarity of the 5 strongly argues that they are asexual/clonal/mitotic derivatives of a common ancestor that dispersed widely. An outstanding question is whether L is an *obligate asexual*, versus a *facultative sexual* for which asexual growth has been especially successful, while admitting occasional sexual offshoots. We examined this question via a suite of numerical simulations (detailed in `global_thaps_clones/scripts/larrys/asex/asex.pdf`), which all point to *obligate asexuality* as the most likely explanation for the observed data.

The fundamental observation underlying these simulations is the following. While it is of course theoretically possible that random genetic drift explains the spread of the L-genotype, it seems far more likely that it harbors some genetic advantage that powered the spread. Furthermore, that advantage must be *confined* to mitotic offspring, for otherwise it would have spread (via meiosis/facultative sexual reproduction) from the clonal genotype in question into the remainder of the population, preventing L from ever having gained global prominence. Obligate asexuality is both the simplest model explaining mitotic confinement and the one most easily seen to agree with our observational data: as shown below, facultative sex results in unexpected and unobserved characteristics in the non-L population structure. It has often been noted in the literature that clonal strains may enjoy an advantage from potentially complex *combinations* of alleles that serendipitously produce an especially fit genotype, combinations that are likely to be disrupted by meiosis, which results in a “regression to the mean” phenotype. Such complex combinations provide an example of mitotic confinement within a facultative sexual species–if the constituent alleles are advantageous in combination, but nearly neutral in isolation, then even though the individual alleles can “leak” into the population at large via facultative sexuality, the advantageous combination does not, hence confining the advantage to the mitotic lineages. (For simplicity, this is called a “complex trait” below.) A rare, advantageous recessive allele provides an even simpler example of mitotic confinement within a facultative sexual. If a homozygote appears (say, by inbreeding or gene conversion), it is advantaged, as are its mitotic offspring, but because the recessive allele is rare, sexual reproduction overwhelmingly produces heterozygotes, wherein the allele is effectively neutral.

Our final observation is that mitotic expansion driven by a confined advantage in a facultative sexual leads to a sharp change in the genetic structure of the remainder of the population. The reason for this is simple: every clonal cell undergoing meiosis injects its (post-recombination) haplotypes into the population. If none produce viable offspring, the clonal genotype is effectively asexual, whereas

successful mating, whether selfing or mating with other members of the population, produces genotypes that are recognizably *not* part of the global clonal lineage (the "L-population"), thereby steadily altering the allele frequencies among the non-L population to more closely resemble frequencies in the original clonal L genotype. In particular, assuming a "fully confined" mitotic advantage, i.e., one where the clone's competitive advantage is never offset, this will eventually push positions that are homozygous in the L-genotype to fixation in the non-L population. Likewise, positions that are heterozygous in the L-genotype will be pushed to 50% frequency in the non-L population (but in a Hardy-Weinberg mixture of states, not purely heterozygous). Somewhat surprisingly, this happens genome-wide, not just at loci genetically linked to the alleles that provide advantage to the L-genotype.

One important consequence of this model is that a homozygous advantageous recessive allele is not fully confined. As the L-clone becomes a sizeable fraction of the total population, the sexual injection of L-haplotypes into the remainder of the population raises the allele frequency of the recessive there, enabling the emergence of homozygous recessives on different genetic backgrounds, which then compete with the L-clones, and by assumption have the same advantage. Furthermore, the descendants of L-clones that commit to gametogenesis exit the L-clonal portion of the population, whereas descendants of non-L-genotypes that carry the recessive allele homozygously remain in the non-L portion of the population. This imbalance means that clonal growth of the L-genotype will *peak, then fall to extinction* in this scenario (interestingly, after having catapulted a recessive allele towards fixation). A similar trajectory may befall L-clones carrying a "complex combination" of alleles–provided the synergistic combination isn't too complex, it may be reconstituted on various genetic backgrounds. In the main text we focused on the recessive model, since it seems both the more intuitive and the more likely, biologically, but note that the extensive and recognizable genome-wide distortion of the allele frequency spectrum of the non-L sub-population is intrinsic to both models of facultative sexual reproduction, and that the observed H-isolates are *not* consistent with the predicted non-L population in either case.

Our simulations provide quantitative support for the intuitions presented above. In all simulations, L-isolates represented an extremely small starting proportion of the population. Simulations represent only relative growth rates within a fixed, finite population. We do not consider complex ecological factors such as blooms, seasonal changes or global circulation. For convenience, the models are parameterized in terms of characteristics of sexual and asexual reproduction (frequency of each, number of offspring, etc.), but these are interconvertible with "selection coefficients" and similar parameters more typically seen in population genetic models. Parameter estimates are based on literature values when available. The maximal rate of sexual reproduction in the field, estimated for pennate diatoms, is about once every 2 years[6, 7]. In this typical life history of diatoms, asexual reproduction would amplify specific genotypes that would be regularly eroded by sexual reproduction.

With respect to reproductive mode only, obligate asexuals would have an exponential growth advantage over sexually reproducing individuals, and would sweep the population in 100 to a few hundred years because mitosis is quicker than meiosis, fertilization, and zygote development combined (Fig. S10A, blue curve). If a reduced rate of sexual reproduction is assumed to be the result of a (mitotically confined) complex trait in the L-isolates, L-genotypes will be maintained in the population at stable intermediate frequencies (Fig. S10A, solid green and red curves). However, as discussed above, when alleles that are identical by descent from L are tracked in the non-L population, we find that growth in L-genotypes drives the non-L population's allele frequencies. Homozygous L-alleles go to fixation while each allele of a heterozygote rises to a 50% frequency (Fig. S10A, dashed and dotted curves). Consequently, there would be no private SNPs in individuals with non-L genotypes, which is very different from what we observe in the H-isolates. (The L-genotype does not rise to fixation in these scenarios since the large L population is continually creating sexual offspring that are recognizably non-L, and which also reproduce.)

If the rate of sexual reproduction in L-individuals is assumed to be ten-fold lower than non-L due solely to a homozygous recessive Mendelian (single-locus) trait, the L-genotype rises to temporary dominance lasting about 30 years (Fig. S10B, red curve). The subsequent collapse of L is again driven by the sexual offspring of L-clade members, which push the advantageous recessive allele into the non-L population to such an extent that homozygous non-L individuals become common (Fig. S10B, orange curve) and outcompete the L-genotype.

The 30-year width of the peak in Fig. S10B is the same time span over which the L-isolates were collected. Given the many parameters (environmental variability, competition, natural selection, etc.) missing from our models, it seems highly improbable that we would have sampled this inbred clade during the specific time period when it was globally dominant. Furthermore, the assumed factor of ten reduction in sexual reproduction rate is perhaps unrealistic; raising the relevant parameter (*bx*) by a factor of 2 to 4 delays the transition by a few centuries, but more importantly reduces the height of the L-clade's peak; i.e., the advantageous recessive allele sweeps to fixation without the L-clade ever reaching a majority of the population. Thus, it is difficult to reconcile any of the facultative sexual reproduction model scenarios with having sampled the L-genotype in 5 of 7 locales.

In summary, growth of the simulated clonal genotype seems to imply that the L-clade came to prominence through what we have called a "confined mitotic advantage." Global growth could be possible if the L-isolates were facultatively sexual, but the simplest form of this (advantageous Mendelian recessive) is incapable of *sustained* high clonal population levels, and, while both models predict coexistence with non-L genotypes, neither model is compatible with non-L genotypes that are anywhere near as genetically distinct as the observed H-isolates. In contrast, inbreeding, loss of heterozygosity, and concomitant conversion to obligate asexuality are sufficient to explain the L-genotype's global growth, and on a relevant time scale. Unfortunately, this model does not predict coexistence with the H-isolates. Given the extremely simplistic nature of the model, however, we don't think that should be a surprise; geographic isolation, local adaptation and many other factors outside the scope of our model are likely to be relevant and important. In short, the most parsimonious explanation is that the L-isolates are an obligate asexual lineage.

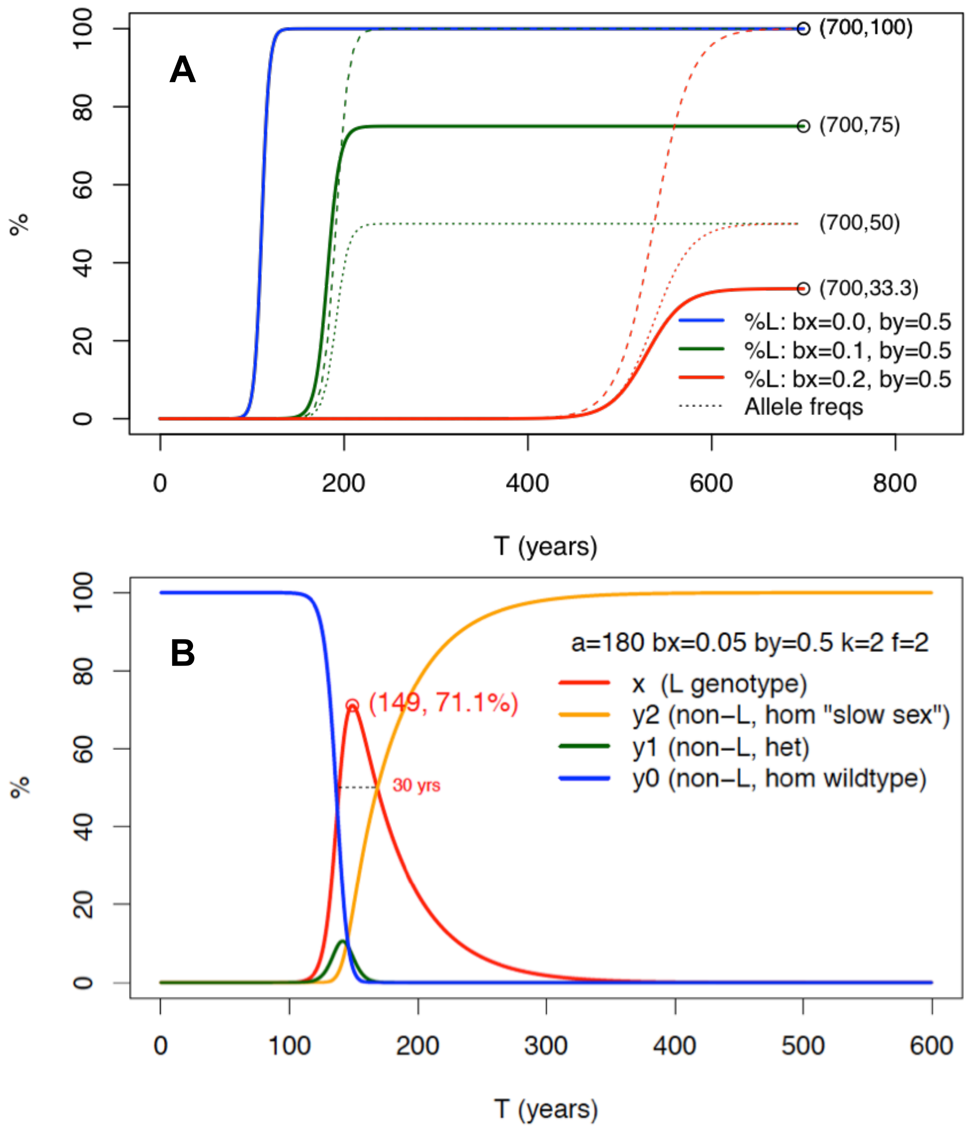

Figure S10. A) Simulated frequencies of L-genotypes and L-alleles over time under low rates of sexual reproduction in L-isolates when that rate is assumed to be dictated by a "complex genetic trait." Model parameters include: initial proportion of L-gentoypes (p_0) = $1{\times}10^{-12}$; number of asexual (mitotic) divisions (a) = 180 per year for both L and non-L individuals; ratio of time required for sexual reproduction (meiosis/zygote development) compared to asexual division (k) = 2; viable offspring per gametic cell (f) = 2; rate of sexual reproduction in L-individuals = bx per year (e.g., 0.1 means once every 10 years); rate of sexual reproduction in non-L ("wild type") individuals = by per year (0.5 means every other year). Solid lines are the frequencies of L-genotypes in the population arising mitotically when the rate of sexual reproduction is varied from bx = 0 (obligate asexuality) to bx = 0.2. Dashed and dotted lines (red and green) follow single alleles through the non-L population that are identical by descent from L and were either homozygous in L (dashed lines) or heterozygous in L (dotted lines). B) Simulated genotype frequencies when the rate of sexual reproduction of L-individuals is assumed to be lowered by a homozygous recessive Mendelian trait. Parameters p_0, a, k, f, and by of the model are as in panel A; rate of sexual reproduction in L-individuals and later homozygous recessives (bx) = 0.05 per year.

9 SNP-based Tree

Approximately half of SNPs are shared among multiple isolates, and, given that fewer that 2% of positions are called SNPs in any strain, shared SNPs are more likely to represent shared ancestry than independent mutation. The sharing pattern is well-captured by a tree structure.

In the tree shown in Figure 3 (main text), internal branch lengths are the numbers of refined SNPs shared by all isolates descendant from that branch of the tree. Terminal branch lengths are the numbers of "private" refined SNPs, that is, those found only in that isolate. The tree topology was chosen by a parsimony criterion: the tree was rooted at the common ancestor of all seven isolates, and we selected each split to maximize the number of SNPs shared within each subtree/minimize sharing across subtrees. Initially, the most significant uncertainty in the tree was the placement of CCMP 1014. Separation of it from the other 6 isolates at the highest level in the tree increased the number of cross-subtree SNPs by only about 10%, but visual inspection favors the L-/H-clade separation and a bootstrap test confirmed that the 10% increase is a statistically significant difference—the 1-6 split was worse than the L-/H-clade split shown in Figure 3 in each of at least a thousand bootstrap replicates. We believe that technical differences in the 1014 data (lower coverage and lower read quality scores), as opposed to biological differences, are the primary drivers of its placement apart from the other L-isolates, but the bootstrap test firmly supports its placement in the L-clade. Other than this technically-driven separation of CCMP 1014 from the other four L-isolates, the differences among them are so slight that the particular 4-branch subtree connecting them may not be statistically significant; all other tree splits certainly are.

Refining SNPs for low coverage, low non-reference allele counts, tri-allelic positions, and/or discordant non-reference alleles in different strains removed 6,496 questionable heterozygous positions from the list (Section 3). Such low frequency of tri-allelic positions (< 2% of total SNPs if all 6,496 are tri-allelic) also reinforces the assumption that all 7 strains are diploid (with possible duplicated genomic regions). Cross-isolate sharing patterns on the remaining heterozygous positions define Figure 3, and that dendrogram captures 82% of refined SNP positions, while 18% of SNPs have sharing patterns that are inconsistent with the tree. The majority of SNPs discordant with this tree are shared by exactly 6 isolates excluding either CCMP 1014 (≈16K such positions; plausibly 7-way sharing masked by low coverage/high error rate in CCMP 1014) or excluding one of the H-isolates (≈12K positions absent from CCMP 1013 alone and ≈15K absent from CCMP 3367 alone; plausibly 7-way sharing of HWE population-level polymorphisms that happens to be homozygous in the individuals we sequenced). Another source of discordance is the result of hemizygous deletion, especially within the L-clade. Repeating this analysis using the SAMtools SNP calls (unrefined) finds fewer shared/more discordant positions, but the inferred tree topology is the same (data not shown).

See `global_thaps_clones/scripts/larrys/shared-snps/` for details and R code for this analysis.

References

1 Armbrust, E. V. *et al.* The genome of the diatom *Thalassiosira pseudonana*: Ecology, evolution, and metabolism. *Science* **306**, 79-86, doi:10.1126/science.1101156 (2004).

2 Abyzov, A., Urban, A. E., Snyder, M. & Gerstein, M. CNVnator: An approach to discover, genotype and characterize typical and atypical CNVs from family and population genome sequencing. *Genome Res.* **21**, 974-984, doi:10.1101/gr.114876.110 (2011).

3 Li, H. & Durbin, R. Fast and accurate long-read alignment with Burrows-Wheeler transform. *Bioinformatics (Oxford, England)* **26**, 589-595, doi:10.1093/bioinformatics/btp698 (2010).

4 van der Auwera, G. A. *et al.* From FastQ data to high-vonfidence variant calls: the genome analysis toolkit best practices pipeline. *Current Protocols in Bioinformatics*, doi:10.1002/0471250953.bi1110s43 (2013).

5 Kleinberg, J. & Tardos, E. *Algorithm Design*. (Addison-Wesley Longman Publishing Co., Inc., 2005).

6 Holtermann, K. E., Bates, S. S., Trainer, V. L., Odell, A. & Armbrust, E. V. Mass sexual reproduction in the toxigenic diatoms *Pseudo-nitzschia australis* and *P. pungens* (Bacillariophyceae) on the Washington coast, USA. *J. Phycol.*, doi:10.1111/j.1529-8817.2009.00792.x (2010).

7 D'Alelio, D. *et al.* The time for sex: A biennial life cycle in a marine planktonic diatom. *Limnol. Oceanogr.* **55**, 106-114 (2010).
